# Green-synthesized ZnO–MnO nanocomposite as a potent antimicrobial and antibiofilm agent: protein leakage mechanism and synergistic interaction with cefotaxime

**DOI:** 10.3389/fmicb.2026.1851865

**Published:** 2026-05-22

**Authors:** Mohammed S. Abdulrahman, Nasir A. Ibrahim, Amr H. Hashem, Ebrahim Saied, Fathy M. Elkady, Ahmed Abdel Tawab, Bahaa M. Badr, Samy Selim, Ashwag Jaman Alzahrani, Hassan H. Alhassan, Nosiba S. Basher, Sulaiman A. Alsalamah, Fahd A. Nasr, Mohammed Aufy

**Affiliations:** 1 Department of Microbiology and Immunology, Faculty of Pharmacy (Boys), Al-Azhar University, Cairo, Egypt; 2 Department of Microbiology and Immunology, Faculty of Pharmacy, Menoufia National University, Menoufia, Egypt; 3 Department of Biology, College of Science, Imam Mohammad Ibn Saud Islamic University (IMSIU), Riyadh, Saudi Arabia; 4 Department of Botany and Microbiology, Faculty of Science, Al-Azhar University, Cairo, Egypt; 5 Department of Microbiology and Immunology, Faculty of Medicine, Al-Azhar University, Cairo, Egypt; 6 Department of Basic Medical and Dental Sciences, Faculty of Dentistry, Zarqa University, Zarqa, Jordan; 7 Department of Medical Microbiology and Immunology, Faculty of Medicine, Al-Azhar University (Assiut Branch), Assiut, Egypt; 8 Department of Clinical Laboratory Sciences, College of Applied Medical Sciences, Jouf University, Sakaka, Saudi Arabia; 9 Department of Biological Sciences, Faculty of Science, University of Jeddah, Jeddah, Saudi Arabia; 10 Department of Pharmaceutical Sciences, Division of Pharmacology and Toxicology, University of Vienna, Vienna, Austria

**Keywords:** antibiofilm activity, antimicrobial activity, characterization, green biosynthesis, nanocomposites

## Abstract

**Introduction:**

Multidrug-resistant (MDR) *Acinetobacter baumannii* infection is a major global public health concern due to its rapid emergence and increasing resistance to conventional antimicrobial agents. Therefore, the development of novel and environmentally friendly antimicrobial alternatives is urgently needed.

**Methods:**

In this study, zinc oxide–manganese oxide bimetallic nanocomposite (ZnO–MnO BNC) was successfully synthesized using potato peel extract through a green synthesis approach. The synthesized nanocomposite was characterized using UV–Vis spectroscopy, FTIR, TEM, and XRD analyses. Its biological activities, including cytotoxic, antibacterial, antibiofilm, bactericidal, and synergistic effects with cefotaxime, were evaluated.

**Results:**

UV–Vis spectroscopy showed characteristic absorption peaks at 300 and 380 nm, confirming nanocomposite formation. FTIR analysis identified the functional groups responsible for bioreduction and stabilization, along with Zn–O and Mn–O bonds. TEM analysis revealed nanosized particles ranging from 10 to 60 nm with an average size of approximately 35 nm, while XRD confirmed the crystalline structure of ZnO and MnO phases. Biological evaluation demonstrated moderate toxicity toward WI-38 normal cells (IC50 = 168.34 μg/mL) and strong anticancer activity against Hep-G2 and MCF-7 cell lines, with IC50 values of 30.56 and 56.1 μg/mL, respectively. The ZnO–MnO BNC exhibited potent antibacterial activity against MDR *Acinetobacter baumannii* clinical isolates, producing inhibition zones up to 20.33 ± 0.58 mm and minimum inhibitory concentrations ranging from 32 to 512 μg/mL. Additionally, the nanocomposite showed significant antibiofilm activity (up to 62.99%), rapid bactericidal kinetics, time-kill efficacy, and pronounced protein leakage (up to 73.46%). Synergistic interaction with cefotaxime further enhanced antibacterial activity.

**Discussion:**

The findings highlight the potential of biogenic ZnO–MnO BNC as an eco-friendly nanoplatform with promising antimicrobial and anticancer applications. The strong antibacterial, antibiofilm, and synergistic activities suggest its possible use as an alternative strategy to combat MDR bacterial infections.

## Introduction

The rapid advancement of nanoscience has reshaped modern biomedical and environmental technologies by enabling the development of materials with tunable physicochemical properties at the nanoscale (Tovar-Lopez, 2023). Metal oxide nanoparticles (NPs), in particular, have gained considerable attention due to their catalytic activity, redox behavior, optical properties, and broad-spectrum biological potential. However, conventional fabrication strategies often rely on high-energy inputs, toxic solvents, and synthetic stabilizers, raising concerns regarding environmental safety and long-term biomedical compatibility (Mathur et al., 2024). In response to these limitations, biologically mediated synthesis has emerged as a sustainable alternative. This approach utilizes naturally occurring biomolecules to drive NPs formation under mild conditions, eliminating the need for harsh reagents (Kirubakaran et al., 2026). Among biological systems, plant extracts are particularly advantageous because they contain diverse phytochemicals capable of simultaneously acting as reducing, chelating, and stabilizing agents. Flavonoids, polyphenols, organic acids, and reducing sugars contribute to controlled nucleation and growth processes, resulting in structurally stable and surface-functionalized nanomaterials (Rizvi et al., 2022; Singh et al., 2023).

Unlike single-component NPs, metal oxide NCs have recently attracted increased research interest due to their synergistic physicochemical behavior (Gupta et al., 2018; Gomaa et al., 2024). When two semiconducting oxides are combined, interfacial charge transfer phenomena may occur, leading to enhanced redox cycling, improved electron mobility, and increased reactive oxygen species (ROS) generation (Balapure et al., 2024). These characteristics are especially relevant in biomedical applications, where oxidative stress plays a critical role in antimicrobial and anticancer mechanisms. Zinc oxide remains one of the most extensively studied semiconductor oxides due to its wide band gap, chemical stability, and recognized biocompatibility (Mandal et al., 2022). It has been incorporated into wound-healing materials, antimicrobial coatings, UV-protective formulations, and drug delivery systems. Meanwhile, MnO exhibits versatile redox states and catalytic behavior, making it suitable for oxidative stress modulation and enhanced antibacterial performance. Integrating ZnO with MnO within an NC framework may therefore provide complementary electronic interactions and superior biological activity compared to individual oxides (El-Moslamy et al., 2024; Selim et al., 2025a).

Potato peel extract has recently attracted attention as an eco-friendly and value-added biological resource for NPs synthesis. As an abundant agricultural byproduct, potato peel represents a sustainable raw material rich in bioactive phytochemicals (Ijaz et al., 2024). It contains significant amounts of phenolic compounds (such as chlorogenic acid and caffeic acid derivatives), flavonoids, reducing sugars, starch residues, organic acids, and trace proteins. These constituents play a dual role during NPs formation: they act as natural reducing agents that convert metal precursor salts into their corresponding oxide NPs and simultaneously function as stabilizing and capping agents that prevent excessive aggregation (Szczyglewska et al., 2023). The high antioxidant capacity of potato peel extract enhances its reducing efficiency, enabling controlled nucleation and growth of nanostructures under mild reaction conditions without the need for hazardous chemicals or high-energy inputs. Additionally, the presence of polysaccharides and phenolic hydroxyl groups contributes to improved surface functionalization, which may enhance colloidal stability and biological interactions. Utilizing potato peel extract, therefore, not only supports green chemistry principles but also promotes waste valorization and circular bioeconomy approaches by transforming agro-industrial residues into high-value nanomaterials (Khanal et al., 2024).

The ever-rising prevalence of the MDR phenotype is making the Gram-negative bacterial infections a serious and global public health challenge (Kaur et al., 2024). Also, the quick expansion of pathogenic bacteria with antimicrobial resistance (AMR) phenotype is associated with an elevated mortality rate (Tabassum et al., 2024). *Acinetobacter baumannii* is an ubiquitous microorganism observed in water, soil, plants, animals, and humans. It is an opportunistic bacterium with the ability to cause various illnesses, such as pneumonia, bacteremia, wound infections, urinary tract infections, and meningitis, in susceptible patients (Eissa, 2024). Unregulated antimicrobial agent usage is among the factors associated with the increased incidence of MDR *A. baumannii* phenotype-associated infections. Immunocompromised patients with a serious infection caused by extensively drug-resistant *A. baumannii* showed poor prognosis and elevated mortality rate, making its treatment challenging (Shi et al., 2024). To control these MDR bacterial infections, research is required to find suitable and effective antimicrobial alternatives, such as the use of immunostimulants, enzymes, and organic acids (Girma et al., 2024; Yakoup et al., 2024). Also, the application of nanostructured systems is specifically obvious in analytical and pharmacological fields. Accordingly, NPs formulations represent a valuable opportunity for the eradication of MDR pathogens (Virzì et al., 2024). Moreover, multi-metallic NPs formulation demonstrated remarkable and promising activity against AMR pathogens due to their unique physiochemical properties, such as shape, size, surface charge potential, and surface area to volume ratio (Tasnim et al., 2023). These multifunctional properties, associated with the release of metal ions, induction of ROS production, interaction with subsequent disruption of microbial cell membranes, dysfunction of cellular biomolecules, and achievement of DNA damage (Kalakonda et al., 2024; Tyutrina et al., 2024).

Studies investigating plant-mediated fabrication of ZnO–MnO heterostructures using agro-industrial waste extracts, such as potato peel, are limited, and the connection between their structural crystallinity, interfacial coupling, and multifunctional biological activity has not been fully clarified despite the growing interest in green NC synthesis. By creating a sustainable method for synthesizing ZnO–MnO BNC using potato peels and thoroughly assessing their biological performance, the current study fills these gaps. The promise of ZnO–MnO BNC as a viable nanoplatform for tackling the increasing problem of AMR is highlighted by this integrative strategy, which combines environmentally friendly synthesis with mechanistic biology research. This study aims to develop a sustainable, eco-friendly, plant-assisted route for the synthesis of binary ZnO–MnO NC and to comprehensively investigate their structural and physicochemical characteristics. Furthermore, this work seeks to evaluate the biological performance of the synthesized NC, with particular emphasis on their cytotoxic and anticancer activities, as well as their antibacterial efficacy against MDR *A. baumannii* clinical isolates.

## Materials and methods

### Chemicals

Analytical-grade zinc acetate dihydrate (Zn(CH₃COO)₂·2H₂O, ≥99%) and manganese nitrate hexahydrate (Mn(NO₃)₂·6H₂O, ≥99%) were used as metal precursors without further purification. All aqueous solutions were prepared using double-distilled water throughout the experiments.

### Biosynthesis and characterization of ZnO–MnO BNC

#### Preparation of potato peel extract

Potato peels were collected from local market sources in Giza, Egypt. The collected peels were thoroughly rinsed several times with distilled water to remove surface impurities, followed by air drying at room temperature. The dried material was finely ground into powder form. 10 g of the powdered peels were mixed with 100 mL of double-distilled water in a 250-mL Erlenmeyer flask and heated at 80 °C for 60 min under continuous stirring. After cooling to ambient temperature, the extract was filtered through Whatman No. 1 filter paper to remove solid residues. The filtrate was stored at 4 °C and used within 48 h for NPs synthesis (Girigoswami et al., 2024).

#### Green synthesis of ZnO–MnO BNC

Equal molar aqueous solutions (1 mM) of zinc acetate dihydrate and manganese nitrate hexahydrate were prepared separately and then mixed in a 1:1 volume ratio to obtain the precursor solution. Subsequently, 20 mL of potato peel extract was added dropwise to 120 mL of the mixed metal precursor solution under continuous magnetic stirring at room temperature. The reaction mixture was maintained under stirring for 1 h. A gradual color change from pale yellow to light brown indicated the reduction and formation of metal oxide nanostructures. The resulting suspension was centrifuged at 15,000 rpm for 10 min to collect the precipitated NC (Selim et al., 2025b). The obtained solid was washed repeatedly with ethanol, followed by deionized water to remove unreacted biomolecules and residual ions. The purified material was dried at 100–120 °C for 12 h and then gently ground into fine powder for further characterization (Somu et al., 2019).

#### Characterization techniques

The optical absorption properties of the synthesized ZnO–MnO BNC were examined using a JENWAY 6305 UV–vis spectrophotometer (Staffordshire, UK) over a wavelength range of 200–800 nm. Fourier transform infrared (FTIR) analysis was carried out using a Cary 660 FTIR spectrometer (Agilent Technologies, USA) employing the KBr pellet technique within the spectral range of 400–4,000 cm^−1^ to identify the functional groups involved in NPs reduction and stabilization. The crystalline phase and structural properties were analyzed using a Shimadzu XRD-6000 diffractometer (Shimadzu Scientific Instruments, Japan) equipped with Cu Kα radiation (*λ* = 1.5406 Å), operated at 40 kV and 30 mA, with a scanning range of 2*θ* = 10°–80°. The crystallite size was calculated using the Debye–Scherrer equation (Basak et al., 2022): D=Kλ/(*β*cosθ), where *K* = 0.9, λ = 1.5406 Å, β represents the full width at half maximum (FWHM) in radians, and θ is the Bragg angle.

Morphological characteristics, particle size distribution, and lattice fringes were investigated using transmission electron microscopy (TEM) (JEM-2100 Plus, JEOL Ltd., Japan) at an accelerating voltage of 200 kV. The hydrodynamic diameter and colloidal stability of the NC suspension were evaluated using the dynamic light scattering (DLS) assay (Zetasizer Nano ZS, Malvern Instruments Ltd., UK).

#### Evaluation of ZnO-MnO BNC cytotoxicity and anticancer activity

The cytotoxicity of ZnO–MnO BNC was evaluated toward WI-38 normal cell line using the 3-(4,5-dimethylthiazol-2-yl)-2,5-diphenyltetrazolium bromide (MTT) assay (Van de Loosdrecht et al., 1994). The anticancer activity was also investigated against the cancerous Hep-G2 and MCF-7 cell lines, which were obtained from the American Type Culture Collection (ATCC). The optical density (OD) of the samples was measured at a wavelength of approximately 560 nm. The following Equations 1 and 2 were then used to determine the total cell count and the proportion of viable cells.

$$\text{Viability}\%=\frac{\text{Sample}\;\mathrm{OD}}{\text{Control}\;\mathrm{OD}}\times 100\;$$ (1)

Cytotoxicity%=100−Viability% (2)

#### Isolation and identification of *A. baumannii*

In this study, various clinical specimens were collected under aseptic conditions and inoculated into MacConkey’s agar, appropriate for isolation of *A. baumannii*, followed by incubation for 24 h at 37 °C; *A. baumannii* ATCC 17978 (Ab_17978_) was included as a reference strain. The lactose non-fermenting isolates were then phenotypically identified according to Valcek et al. (2023). The motility, type of Gram reaction, capsular staining, and ability to grow at 42 °C and 44 °C were evaluated. Biochemically, oxidase, catalase, ornithine decarboxylase, and tryptophanase enzyme production were assessed. Also, culture characteristics based on their growth behavior on triple sugar iron (TSI) agar and Simmons’s citrate agar were inspected (Rajmichael et al., 2025).

Genotypic confirmation of *A. baumannii* was established by detection of the species-specific *bla*_OXA-51-like_ gene after bacterial genomic DNA extraction following the cell lysis thermal method (Adeyemi et al., 2025). The PCR reaction mixture contained 12.5 μL of Taq master mix (Qiagen, Germany), 1 μL of the extracted DNA, and 1 μL of specific primer, 5’-CTAATAATTGATCTACTCAAGTTAC-3′ and 5’-GAATACTCCATTTGAACCARTGG-3′, in a total volume of 25 μL. The applied PCR conditions were as follows: 1 cycle of denaturation at 95 °C for 5 min, 30 cycles of denaturation at 95 °C for 1 min, annealing at 56.5 °C for 45 s, extension at 72 °C for 1 min, followed by 1 cycle of final extension at 72 °C for 10 min. Agarose gel electrophoresis was then conducted for PCR product detection and visualization with the aid of ethidium bromide and a UV transilluminator. The PCR amplified products were compared with a 1,500 bp DNA ladder.

#### Evaluation of antimicrobial susceptibility patterns

The antimicrobial susceptibility of *A. baumannii* for different antimicrobial agents was carried out according to Bauer et al. (1966). Each tested bacterial suspension of 0.5 McFarland standard turbidity (1.5 × 10^8^ CFU/mL) was prepared. Using a sterile cotton swab, the prepared bacterial suspension was regularly spread on Mueller-Hinton agar (MHA). After 10 min, the antimicrobial discs, representing different classes, were placed on the MHA surface, followed by incubation for 24 h at 37 °C. Result interpretation was conducted on the basis of Clinical Laboratories Standards Institute (CLSI, 2023) guidelines (Pierce et al., 2023).

#### Qualitative antimicrobial assay of ZnO–MnO BNC activities

The inhibitory activity of the biosynthesized BNC against MDR biofilm-forming *A. baumannii* isolates was assessed using the well diffusion method. Each tested bacterium inoculum was prepared by growing in tryptic soya broth (TSB) for 24 h at 37 °C, and culture turbidity was maintained equivalent to 0.5 McFarland standard using 0.9% NaCl solution. Swabbing was employed for bacterial culture inoculation onto MHA plates for the confluent lawn of bacterial growth creation. A sterilized cup borer was then used to make wells of 8 mm on the MHA surface. Each well was then filled with 100 μL of either the tested BNC suspension (1,000 μg/mL), potato peel extract, zinc acetate dihydrate [Zn(CH_3_COO)_2_.2H_2_O] (1,000 μg/mL), manganese nitrate hexahydrate (Mn(NO₃)₂·6H₂O) (1,000 μg/mL), colistin solution (100 μg/mL) as positive control, or dimethyl sulfoxide (DMSO) 10% (solvent used for BNC and colistin) as negative control. Following plate incubation for 24 h at 37 °C, the clear zone formed around a well containing BNC was measured and compared with that formed around the well containing the colistin standard solution. The percent inhibition (PI) was consequently calculated as described in Equation 3 (Elkady et al., 2025).

$$\text{Inhibition}\;(\%)=\frac{\mathrm{IZD}\;\text{caused}\;\mathrm{by}\;\mathrm{ZnO}-\mathrm{MnO}\;\mathrm{BNC}}{\mathrm{IZD}\;\text{caused}\;\mathrm{by}\;\text{colistin}\;}\;\;\;X\;\;\;100\;\;$$ (3)

#### Quantitative antimicrobial assay of BNC activities

The quantitative capability of the biosynthesized ZnO–Mn BNC to cease the MDR biofilm-forming *A. baumannii* isolates growth was analyzed using a microtiter plate with the aid of resazurin dye, which indicates the cell viability changed from blue/non-fluorescent form to pink/highly fluorescent state following the chemical reduction caused by bacterial growth. The minimum inhibitory concentration (MIC) of BNC for each tested isolate was determined using a broth microdilution assay (Tümer, 2025). The ZnO–MnO BNC stock concentration was prepared, followed by twofold serial dilution in a microtiter plate using TSB. Additionally, positive and negative controls were included in each plate, followed by aerobic incubation for 18 h at 37 °C. Resazurin (0.015%) was then added, followed by further incubation for 2 h. Finally, the lowest ZnO–Mn BNC concentration showing no bacterial growth (resazurin color remained blue) was considered as the MIC. For evaluation of ZnO–MnO BNC minimum bactericidal concentration (MBC) for different *A. baumannii* isolates, bacterial inoculum in each well containing equal or more than MIC of the tested BNC was subsequently inoculated onto MHA and incubated at 37 °C for 18 h. The lowest concentration of ZnO–Mn BNC, which caused non-observable bacterial growth on the solid medium, was considered as the MBC of the tested ZnO–MnO BNC. Also, the tolerance index for each tested isolate toward the biosynthesized ZnO–Mn BNC was calculated as the MBC/MIC ratio. Finally, the tolerance index value ≤4 indicates a bactericidal effect, while a value >4 correlates to bacteriostatic capability.

#### *Acinetobacter baumannii* biofilm formation in the presence of ZnO–Mn BNC

The quantitative ability of the tested *A. baumannii* isolates for different degrees of biofilm formation, as well as the biosynthesized ZnO–MnO BNC effect, at a sub-growth inhibitory concentration, on their biofilm formation was evaluated following crystal violet (CV) staining assay in 96-well microtiter plates (Elkady et al., 2024). In brief, ZnO–MnO BNC at the ½MIC or ¼MIC was used, and each experiment was performed as a triplet. The wells were separately inoculated with 10 μL of each bacterial suspension of 0.5 McFarland standard and 180 μL of Luria Bertani broth supplemented with 2% glucose (LBG). The BNC suspension in DMSO (10 μL) was added to each well, followed by incubation for 16 h at 37 °C. The well containing BNC untreated bacterial culture, as a positive control, and the well without bacterial inoculation, as a negative control, were included. The LBG broth and the suspended cells were quietly and completely removed, followed by twice washing of each well with phosphate buffer saline (PBS). The 0.4% CV (200 μL) was then added to each well, followed by incubation at 37 °C for 20 min to stain the remaining attached cells. The excess stain was removed, and PBS was used to wash the wells three times to remove the unbound dye. The CV retained by bacterial biofilm was dissolved by the addition of 95% ethanol (200 μL), followed by incubation for 30 min. The OD of the obtained bacterial biofilm suspension was then measured at 620 nm with the aid of an ELISA microtiter plate reader (Multiskan Ascent, Labsystems, Helsinki, Finland). From the untreated experiment, the OD cut-off (OD_c_), that equal the mean OD + 3 standard deviations (SD) for the negative control, was then calculated. Consequently, each tested bacterial isolate was categorized into either strong (in case of OD > 4OD_c_), moderate (for 2OD_c_ < OD < 4OD_c_), weak (OD_c_ < OD < 2OD_c_), and non- (for OD < OD_c_), and biofilm-forming was then recorded. Finally, the change in bacterial biofilm, caused by the biosynthesized ZnO–MnO BNC, was calculated as a percentage change according to Equation 4 (Jang et al., 2023):

$$\begin{array}{l} \text{Biofilm inhibition}\;(\%)=\\ 1-\frac{\mathrm{OD}620\;\text{of cells treated with}\;\mathrm{BNC}}{\mathrm{OD}620\;\text{of untreated cell}}\times 100\; \end{array}$$ (4)

#### Bacterial killing kinetics of ZnO–MnO BNC

For the evaluation of ZnO–MnO BNC bacterial destructive ability toward *A. baumannii* strain, time–kill assay was employed (Kinoan and Katas, 2025). Briefly, normal saline was used to suspend the purified bacterial colonies at 0.5 McFarland standard, followed by their inoculation into TSB and incubation at 37 °C for 2 h. The tested strain was then treated with either the MIC, 2MIC, or 4MIC of the ZnO–MnO BNC, followed by consequent incubation at 37 °C. The OD at a wavelength of 600 nm (OD_600_) was recorded for each suspension at different time intervals (0, 2, 4, 6, 8, and 24 h). The assay was conducted in triplicate, and the untreated bacterial suspension was included as a positive control to determine and compare the viability of bacterial cells.

#### Antibacterial effect of ZnO–MnO BNC/cefotaxime combinations

After separate determination of ZnO–MnO BNC and cefotaxime MIC, the two-dimensional checkerboard titrations following broth microdilution assay were employed for assessment of their possible interaction at different combined formulations (Bellio et al., 2021). In checkerboard titration, the columns contained equal volumes of cefotaxime with twofold dilution along the X-axis, while the rows contained equal volumes of the tested BNC with 2-fold dilution along the Y-axis. The tested bacterial suspension was then added to each well, followed by plate incubation at 37 °C for 24 h. The MIC, in each raw, was determined, and the fractional inhibitory concentration (FIC) and FIC Index (FICI) values were calculated for the tested BNC and cefotaxime according to Equations 5 and 6, respectively.

FIC=MICin combination/MICalone (5)

$$\text{FICI}=\text{FICBNC}+\mathrm{FI}C_{\text{cefotaxime}}$$ (6)

The nature of the combined agents’ interactions was based on the interpretation of the obtained FICI value, which was as follows: <0.5: synergy, 0.5–0.75: partial synergy, 0.76–1.0: additive effect, >1.0–4.0: indifferent, and >4.0: antagonism.

#### Effect of ZnO–MnO BNC on membrane integrity

Protein leakage assay was employed for assessment of ZnO–MnO BNC effect on bacterial membrane integrity and consequent expectation of one of its possible mechanisms of action. Intracellular bacterial proteins, emitted externally after treatment with the tested BNC, indicate their interaction and subsequent disruption of membrane functions. In brief, each tested bacterial suspension, adjusted to 0.5 McFarland standard, with equal volumes, were exposed to the tested BNC suspension doses equivalent to its 0.25, 0.5, 1, or 2MIC. Triton X–100 (1%) was utilized as a positive lysis control, while the experiment without non-destructive PBS was included as a growth control. At suitable incubation intervals, samples were collected, centrifuged, and a colorimetric method based on Bradford assay was used for measurement of the bacterial leaked proteins concentration in the obtained supernatant. The obtained data were normalized based on the findings of the untreated cell supernatant measured under the same conditions and wavelength (Shalini et al., 2024).

#### TEM imaging of ZnO–MnO BNC-treated bacterial cells

Transmission electron microscopy is utilized to examine *A. baumannii*-9 treated with ZnO–MnO BNC. The bacteria are cultivated in broth with and without BNC treatment for 6–24 h at 37 °C. Centrifugation (10,000 *g*, 10 min, 4 °C), pellet washing in PBS, and initial fixation in 2.5% glutaraldehyde for 2 to 4 h come next. Following post-fixation in osmium tetroxide, the cells are embedded in epoxy resin, ultrasectioned to a thickness of 50–70 nm using an ultramicrotome, and dehydrated using a graded ethanol series (30–100%, 10–15 min each stage). The cells were then stained with lead citrate and uranyl acetate for 5–10 min each to improve contrast. Ultrathin sections are investigated via TEM at 80–120 kV to observe NPs adhesion to cell walls, membrane disintegration, cytoplasm leakage, and empty cell interiors suggestive of antibacterial damage (Tiwari et al., 2018).

#### Statistical analysis

The mean (M) values for the three independent replicate observations were calculated based on the average of the data in each experiment. Also, the standard error (SE) was calculated and applied to approximate the correctness of the obtained mean. All experimental findings of triplicated measurements were stated as Mean ± SD of the three observations to provide a clear and concise presentation of research findings. Statistical analysis among the treatment means was determined utilizing one-way analysis of variance (ANOVA), and the significance was considered at *p* < 0.05.

## Results and discussion

### Biosynthesis and characterization of ZnO–MnO BNC

#### UV–vis

**Figure 1:**
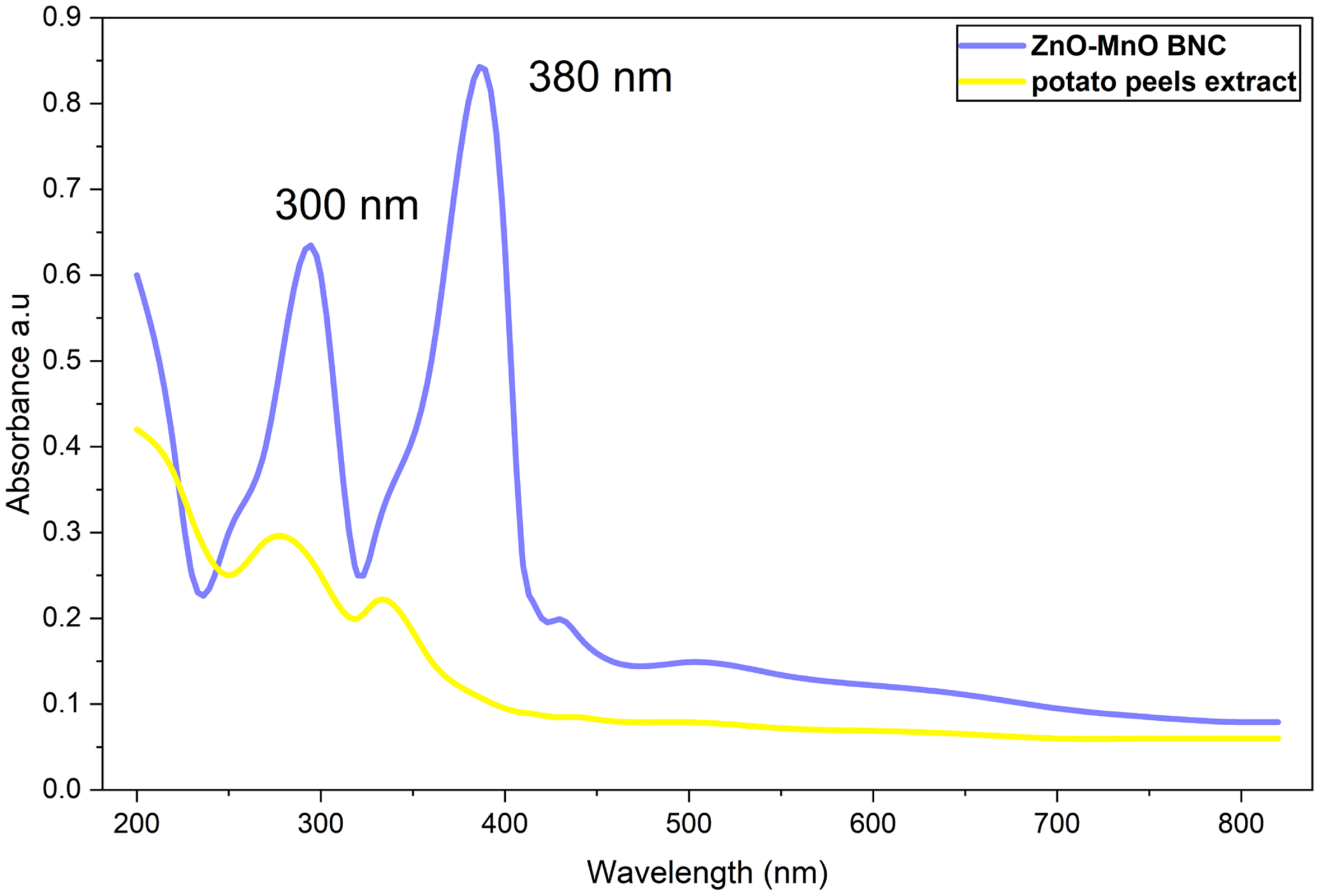
UV–vis spectrum of biosynthesized ZnO-MnO BNC and potato peels extract.

The UV–vis absorption spectrum of the synthesized ZnO–MnO BNC exhibited two prominent absorption bands centered at approximately 300 nm and 380 nm (Figure 1). The weak broad absorption features observed beyond 400 nm may be attributed to surface defect states, charge transfer interactions between ZnO and MnO phases, residual phytochemical capping agents from potato peel extract, and light scattering due to slight NPs aggregation. In contrast, the potato peel extract showed broad and less intense absorption bands in the UV region, which are mainly attributed to phytochemical constituents, such as polyphenols and flavonoids. The strong absorption peak observed at 380 nm corresponds to the intrinsic band-gap absorption of ZnO NPs, arising from electron transition from the valence band to the conduction band (Saravanakumar et al., 2022). The additional absorption band at 300 nm may be attributed to MnO-related electronic transitions and/or interfacial charge transfer within the ZnO–MnO BNC structure. The appearance of well-defined absorption peaks and their increased intensity compared to the plant extract confirm the successful formation of the ZnO–MnO BNC. The characteristic absorption band around 380 nm is consistent with the typical excitonic absorption of ZnO NPs, indicating the preservation of ZnO crystalline structure within the NC (Alamgeer et al., 2023). The slight shift in absorption and the presence of an additional band near 300 nm suggest successful incorporation of MnO into the ZnO matrix, which may alter the electronic structure and band-gap energy due to the metal–metal oxide interaction.

The observed modification in optical behavior is likely associated with electronic coupling between ZnO and MnO phases, defect generation, such as oxygen vacancies, and interfacial charge transfer across the heterojunction structure (Xie et al., 2017). These changes are expected to enhance charge separation efficiency and may contribute to improved biological or catalytic activity of the NC compared to single-metal oxides. Moreover, the significantly lower absorbance of the plant extract beyond 350 nm confirms that the observed absorption peaks are due to NPs formation rather than residual phytochemicals (Pradeep et al., 2022). Recently, El-Moslamy et al. (2024) reported that the UV–vis spectra of MnO–ZnO BNC showed absorption between 350 and 400 nm. Sheema et al. (2025) synthesized ZnO NPs using an aqueous extract of *Curcuma zedoaria*. Al Abboud et al. (2024) prepared ZnO@Au bimetallic NPs (BNPs) via an eco-friendly approach using carboxymethylcellulose (CMC) to formulate the NC. The UV–vis spectra of CMC, ZnO@Au BNPs, and the NC revealed that CMC had no significant peaks, while the BNPs showed two distinct peaks at 371 nm and 523 nm, corresponding to ZnO and Au NPs, respectively. Selim et al. (2025a) reported the green synthesis of Zn–MnO BNC using the fungus *Penicillium rubens*, with UV–vis confirming a top absorption peak at 370 nm. According to earlier studies, transitions caused the Uv–vis absorption spectra of ZnO and MnO NPs to range between 284 and 400 nm (Hoseinpour and Ghaemi, 2018). Martnez-Vargas et al. also showed the fluorescence spectra of myco-fabricated ZnO/MnO BNC with excitation wavelengths of 365, 369, and 371 nm (Martínez-Vargas et al., 2019). Hussein and Mortazavi-Drazkola (2025) reported the green synthesis of ZnO/Au BNC using *Urtica dioica* extract, producing ZnO/Au@UDE NC as an environmentally sustainable alternative to traditional chemical fabrication routes. Similarly, Hasanin et al. (2023) investigated the optical properties of ZnO–CuO and ZnO–CuO/CSC NC using UV–vis spectroscopy. The ZnO–CuO BNC exhibited characteristic absorption peaks at 326, 365, and 410 nm, which were attributed to the formation of a metal oxide heterojunction, confirming successful mycosynthesis in agreement with previously reported findings. In contrast, the ZnO–CuO/CSC NC displayed a distinct narrow band at 275 nm and a broad absorption band around 395 nm, accompanied by noticeable peak shifts. These spectral changes indicated strong interfacial interactions between the metal oxides and the composite matrix. Furthermore, Saied et al. (2026) employed a green biosynthetic strategy to fabricate MnO–silver BNC using *Cucumis melo* peel extract as a natural reducing and stabilizing agent, demonstrating an eco-friendly and sustainable synthesis pathway.

#### FTIR analysis

**Figure 2:**
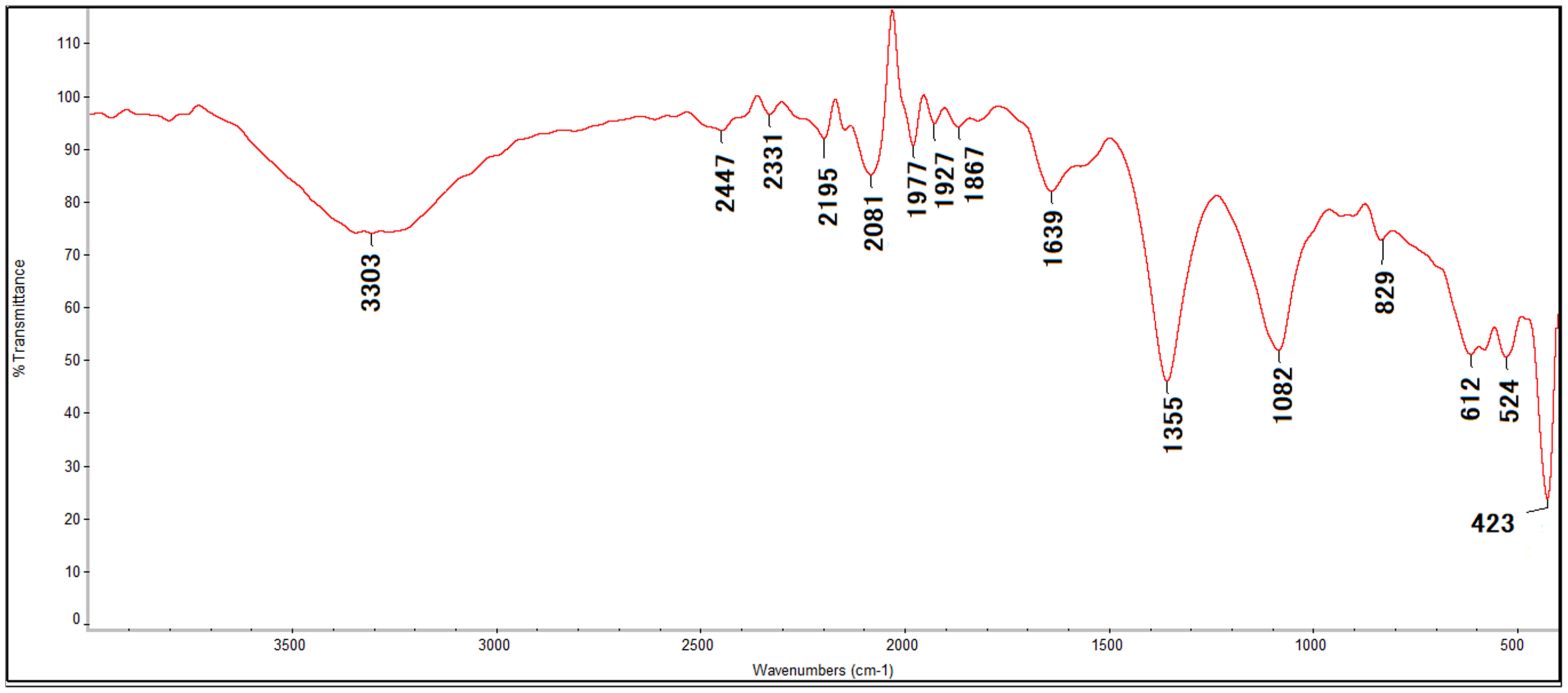
FTIR spectrum of the ZnO–MnO BNC biosynthesized using potato peels extract.

The FTIR spectrum of the synthesized ZnO–MnO BNC exhibited several characteristic absorption bands. The FTIR spectrum of the synthesized ZnO–MnO BNC is presented in Figure 2. The spectrum exhibited characteristic absorption bands at 3,303, 2,447, 2,331, 2,195, 2,081, 1,977, 1,927, 1,867, 1,639, 1,355, 1,082, 829, 612, 524, and 423 cm^−1^, confirming the presence of various functional groups involved in the reduction and stabilization processes, as well as the formation of metal–oxygen bonds within the NC structure. A broad band centered at approximately 3,303 cm^−1^ is attributed to O–H stretching vibrations of hydroxyl groups, indicating the presence of phenolic compounds and adsorbed water molecules (Kainat et al., 2022; Selim et al., 2025b). The absorption band observed at 1,639 cm^−1^ corresponds to C=O stretching or amide I vibrations, suggesting the involvement of proteins or polyphenolic constituents from the potato extract in NPs stabilization (Al Abboud et al., 2024). These functional groups can coordinate with metal ions (Zn^2+^ and Mn^2+^), facilitating nucleation and growth of NPs. The prominent band at 1,355 cm^−1^ may be assigned to C–N stretching or phenolic O–H bending vibrations supports the adsorption of organic phytoconstituents onto the NPs surface, forming a capping layer that enhances colloidal stability (Espina et al., 2022). The peak at 1082 cm^−1^ is attributed to C–O stretching vibrations of alcohols, ethers, or polysaccharides present in the plant extract (Elashmawi and Al-Muntaser, 2021).

Importantly, the absorption bands appearing at 612 cm^−1^ and 524 cm^−1^, along with the strong band at 423 cm^−1^, are characteristic of metal–oxygen (Zn–O and Mn–O) stretching vibrations, confirming the formation of the ZnO–MnO BNC structure (Hakim et al., 2020; Khorsand Zak and Hashim, 2024). However, this interpretation is based on spectroscopic evidence and literature reports, and no direct chromatographic (HPLC/LC–MS) analysis was performed to quantitatively confirm the specific phytochemical composition. Thus, the FTIR results suggest the possible involvement of phytochemicals from potato peel extract in the reduction, functionalization, and stabilization processes.

#### TEM and DLS analysis

**Figure 3:**
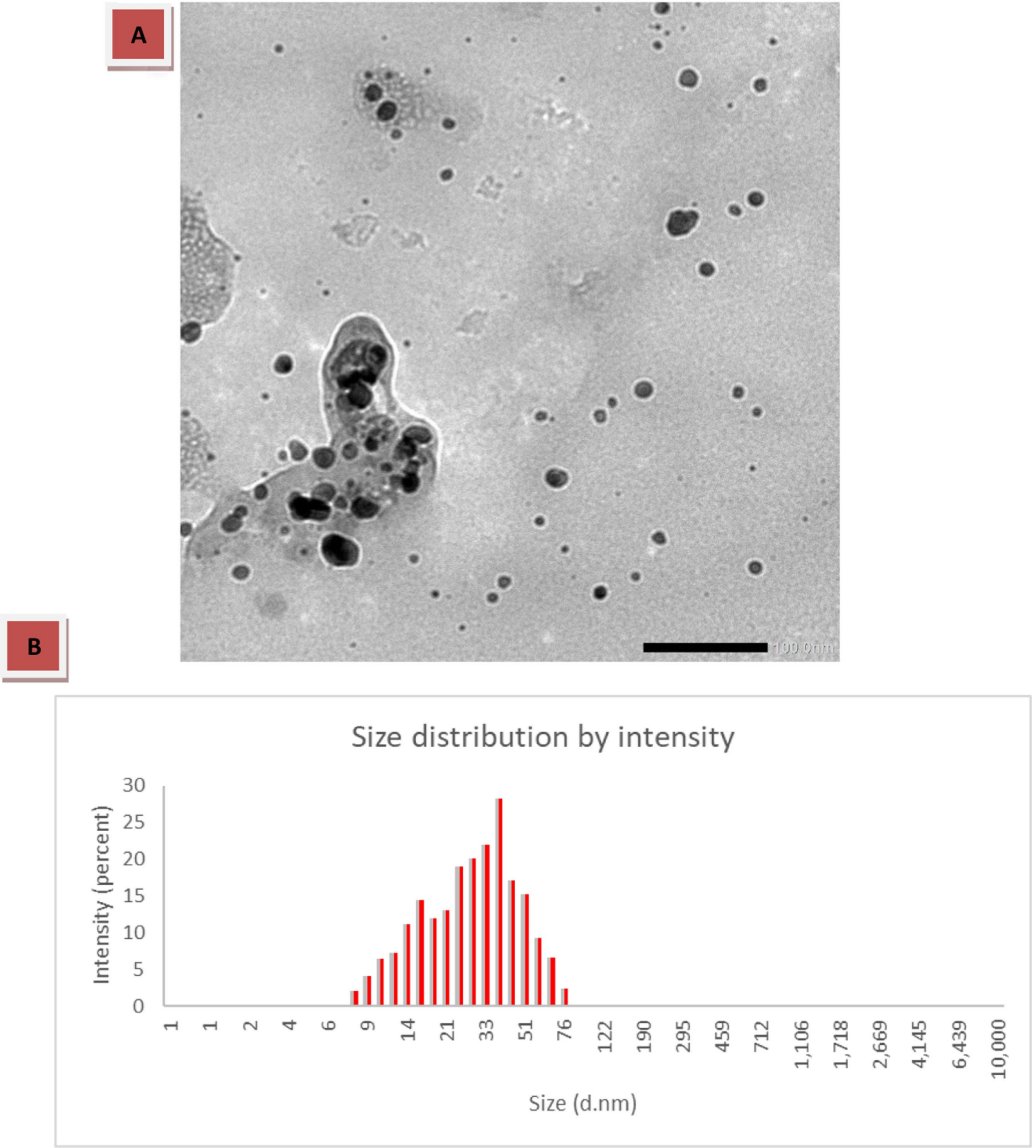
**(A)** TEM micrograph of the synthesized ZnO–MnO BNC showing spherical NPs with partial agglomeration (scale bar = 200 nm). **(B)** DLS size distribution by intensity.

The TEM micrograph (Figure 3) revealed that the synthesized ZnO–MnO BNC particles fall within a size range of approximately 10–60 nm, with an average particle size of about 35 nm. The particles exhibit spherical morphology with slight aggregation in some regions, which is commonly observed in metal oxide nanostructures due to their high surface energy. The observed slight aggregation in TEM images can be attributed to particle–particle interactions during the drying process on the TEM grid (Wilson and Prud'homme, 2021). Additionally, the DLS measurements showed a particle size distribution ranging from 9 to 80 nm, with an average hydrodynamic diameter of approximately 38 nm. The distribution profile indicates a relatively homogeneous colloidal dispersion with no significant secondary peaks corresponding to large aggregates.

The DLS results showed a comparable but slightly higher average size (38 nm) compared to TEM (35 nm). This difference is expected and can be attributed to the fundamental distinction between the two techniques, where TEM measures the physical core size of dried NPs, whereas DLS determines the hydrodynamic diameter of particles in suspension, such as the NPs core, surface-bound phytochemical capping agents, and the surrounding solvation layer (Abraham, 2024). The close agreement between the average sizes obtained from TEM and DLS suggests good dispersion stability and limited agglomeration in the colloidal state. Such nanoscale dimensions (below 100 nm on average) are particularly advantageous for biomedical and antimicrobial applications, as smaller particle sizes enhance surface area, reactivity, and interaction with microbial or cellular membranes (Dolai et al., 2021).

A recent study by Al Abboud et al. (2024) reported the green synthesis of ZnO@Au NC. TEM analysis showed average particle sizes of 15 nm for the BNPs and 25 nm for the NC, while DLS indicated larger hydrodynamic sizes (27 nm and 93 nm, respectively) due to surface-bound phytochemicals and solvation layers, enhancing colloidal stability. Several recent studies have reported diverse morphologies and size distributions for bimetallic and NC systems synthesized via green or alternative approaches. Selim et al. (2025a), demonstrated through TEM analysis, that Zn–MnO BNC exhibited irregular yet nearly spherical particles with sizes ranging from 25.13 to 36.21 nm. In contrast, El-Moslamy et al. (2024) reported comparatively larger dimensions for myco-fabricated ZnO/MnO BNC, with lengths between 132 and 198 nm and widths of 30–62 nm, indicating anisotropic growth behavior. Likewise, Hussein and Mortazavi-Drazkola (2025) synthesized ZnO/Au@UDE NC via a plant-mediated route and observed well-defined spherical and oval morphologies within the 40–50 nm range. Meanwhile, El-Batal et al. (2024) fabricated ZnO–CuO BNPs using gamma irradiation and gum Arabic, obtaining semi-spherical particles with sizes spanning 8.5–70.0 nm and an average diameter of 22.27 ± 1.6 nm. In a similar green synthesis approach, Selim et al. (2025b) prepared MgO–ZnO BNC using *Pluchea indica* leaf extract. TEM analysis revealed predominantly spherical and monodisperse particles ranging from 5 to 35 nm, and a PDI value of 0.31, indicating moderate uniformity. Additionally, Hashem and El-Sayyad (2024) synthesized Ag–ZnO BNPs using pomegranate peel extract and reported spheroidal particles with sizes between 9.5 and 25.2 nm (mean diameter 15.8 ± 1.5 nm), confirming successful nanoscale formation through UV–vis and HR-TEM analyses. Collectively, these findings highlight that the particle size and morphology of BNC strongly depend on the synthesis strategy, reducing agents, stabilizing biomolecules, and fabrication conditions. Variations in phytochemical composition or irradiation methods significantly influence nucleation, growth kinetics, and final nanostructure architecture.

#### XRD analysis

**Figure 4:**
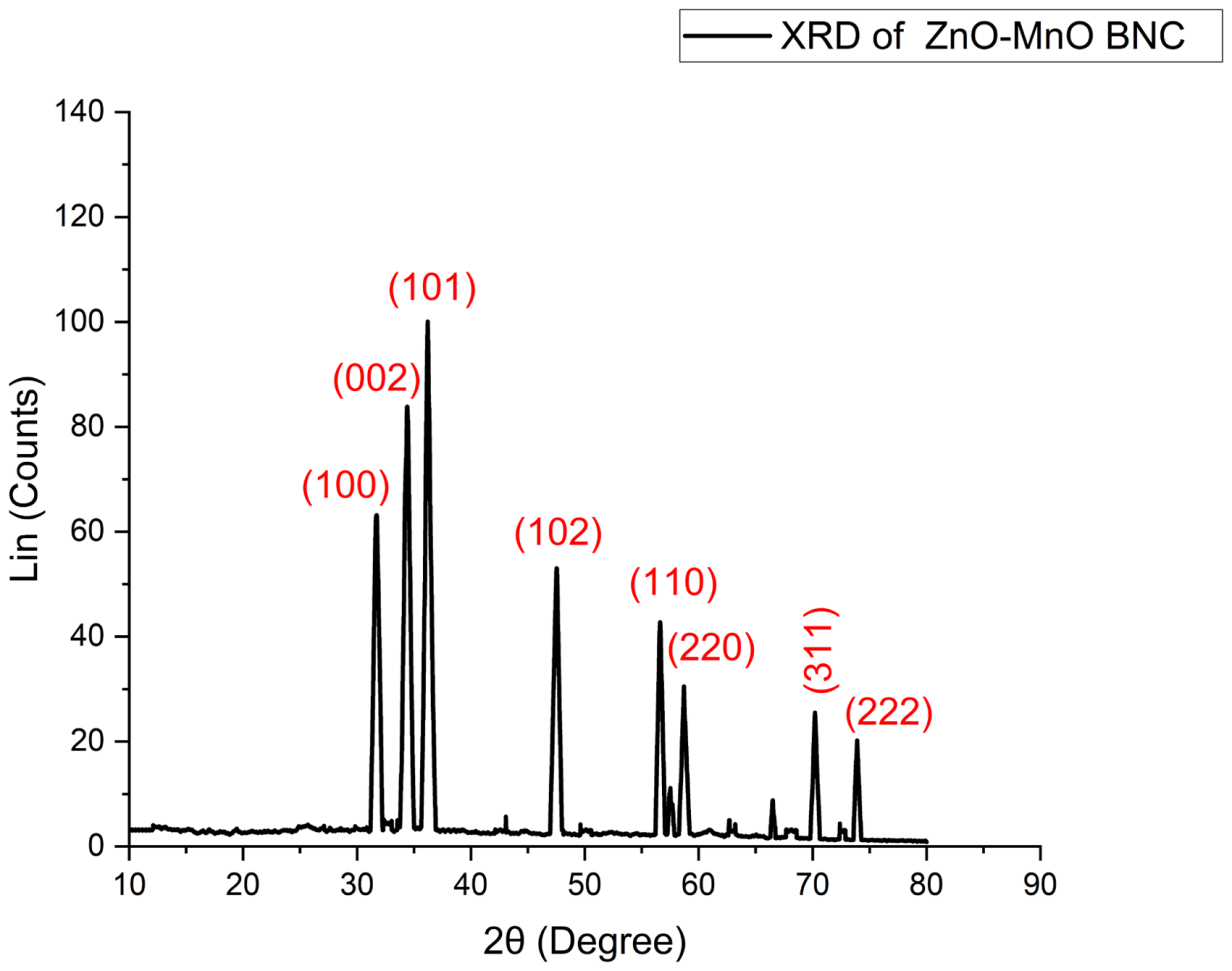
XRD of biosynthesized ZnO–MnO BNC.

The XRD pattern of the biosynthesized ZnO–MnO BNC using potato peel extract exhibited distinct diffraction peaks corresponding to both hexagonal wurtzite ZnO and cubic MnO phases (Figure 4; Table 1). The characteristic reflections of ZnO appeared at 2θ values of 31.7° (100), 34.4° (002), 36.2° (101), 47.5° (102), and 56.6° (110), matching well with the standard JCPDS card no. 36–1,451 (Hernández-Rodríguez et al., 2025). Additionally, secondary diffraction peaks observed at approximately 58.7°, 70.2°, and 73.9° were indexed to the (220), (311), and (222) planes of cubic MnO (JCPDS no. 07–0230) (Pani et al., 2019), confirming the successful formation of a biphasic ZnO–MnO BNC rather than simple Mn doping into the ZnO lattice. The relatively sharp and intense diffraction peaks indicate good crystallinity of the synthesized NC. The higher intensity of the (002) reflection suggests a preferential growth orientation along the c-axis (Umar et al., 2009). No additional impurity peaks were detected, indicating phase purity of the obtained composite. The XRD results confirmed the successful formation of a biphasic ZnO–MnO BNC with well-defined crystalline structures of hexagonal ZnO and cubic MnO. The coexistence of these two crystalline phases is particularly significant from a biological perspective, as it promotes the formation of interfacial heterojunctions between ZnO and MnO domains. The average crystallite size was estimated using the Scherrer equation and was calculated to be approximately 38 nm.

**Table 1:** Indexed XRD diffraction peaks of biosynthesized ZnO–MnO BNC with corresponding Miller indices, crystalline phases, and standard JCPDS reference cards.

2θ (°)	d-Spacing/plane assignment	Miller indices (hkl)	Phase	Crystal structure	JCPDS card No.
31.7	Major characteristic peak	(100)	ZnO	Hexagonal wurtzite	36–1,451
34.4	Major characteristic peak	(002)	ZnO	Hexagonal wurtzite	36–1,451
36.2	Major characteristic peak	(101)	ZnO	Hexagonal wurtzite	36–1,451
47.5	Secondary characteristic peak	(102)	ZnO	Hexagonal wurtzite	36–1,451
56.6	Secondary characteristic peak	(110)	ZnO	Hexagonal wurtzite	36–1,451
58.7	Characteristic MnO peak	(220)	MnO	Cubic	07–0230
70.2	Characteristic MnO peak	(311)	MnO	Cubic	07–0230
73.9	Characteristic MnO peak	(222)	MnO	Cubic	07–0230

Selim et al. (2025a) reported that XRD analysis of ZnO–MnO BNC confirmed the coexistence of two distinct crystalline phases corresponding to MnO (JCPDS No. 81–2,261) and ZnO (JCPDS No. 36–1,451). Similarly, El-Moslamy et al. (2024) demonstrated that the XRD pattern of the myco-fabricated ZnO/MnO NC exhibited a prominent diffraction peak at 2θ = 14° along with weaker reflections at 26° and 32°, indicating high crystallinity. The synthesized material displayed a monoclinic crystalline structure with an average crystallite size of 6.22 nm. In a related study, Hasanin et al. (2023) analyzed ZnO–CuO BNPs and their corresponding NC using XRD. The ZnO phase showed characteristic diffraction peaks at 2θ = 31.66°, 34.26°, 36.06°, 47.56°, 56.43°, and 62.84°, consistent with the hexagonal wurtzite structure (JCPDS No. 36–1,451). Meanwhile, CuO diffraction peaks appeared at 36.06°, 38.76°, 59°, 62.7°, 67.5°, 68.8°, and 73.5°, confirming the monoclinic phase of CuO in agreement with JCPDS No. 48–1,548. Collectively, these studies demonstrate that XRD analysis is essential for confirming phase purity, crystalline structure, and successful formation of BNC, with variations in peak intensity and crystallite size depending on synthesis methodology and precursor interactions.

#### Cytotoxicity and anticancer activity of ZnO–MnO BNC

**Figure 5:**
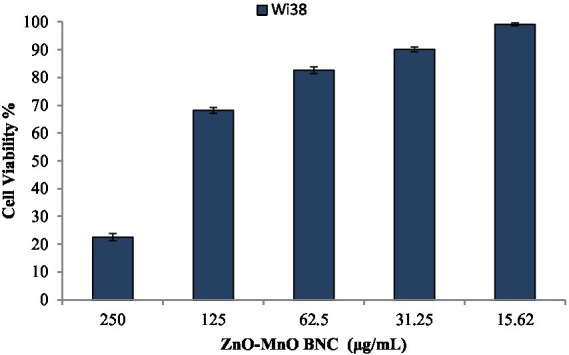
Cytotoxicity of ZnO–MnO BNC toward Wi-38 normal cell line.

The cytotoxicity of the synthesized ZnO–MnO BNC was evaluated against the WI-38 normal human lung fibroblast cell line to determine its biosafety profile and suitability for biomedical applications. The calculated half-maximal inhibitory concentration (IC50) value was 168.34 μg/mL. Generally, if the IC50 value is 90 μg/mL or more, the compound is classified as non-cytotoxic (Ioset et al., 2009). On the other hand, WI-38 cell viability decreased significantly to approximately 22–23%, indicating strong cytotoxic effects. Reducing the concentration to 125 μg/mL improved cell viability to nearly 68%, while further decreases to 62.5 and 31.25 μg/mL resulted in viability values of approximately 82 and 90%, respectively (Figure 5).

**Figure 6:**
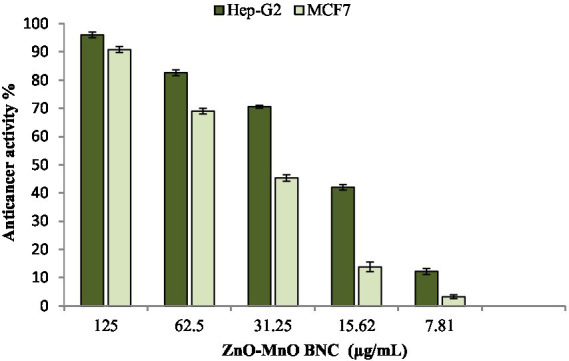
Anticancer activity of ZnO–MnO BNC toward cancerous Hep-G2 and MCF-7 cell lines.

The anticancer potential of the synthesized ZnO–MnO BNC was evaluated against human hepatocellular carcinoma (Hep-G2) and human breast adenocarcinoma (MCF-7) cell lines using a cell viability assay. The obtained results demonstrated a pronounced concentration-dependent inhibitory effect on cancer cell proliferation. Treatment with increasing concentrations of ZnO–MnO BNC resulted in progressive reduction in cellular viability in both cancer cell lines, confirming the cytotoxic efficacy of the synthesized NC. For the Hep-G2 cell line, cell viability decreased from approximately 96.0% at 125 μg/mL to 82.6, 70.6, 42.0, and 12.2% at concentrations of 62.5, 31.25, 15.62, and 7.81 μg/mL, respectively (Figure 6). Similarly, MCF-7 cells exhibited higher sensitivity toward ZnO–MnO BNC exposure, where viability declined from 90.8% at 125 μg/mL to 69.0, 45.3, 13.8, and 3.23% across the same concentration range (Figure 6). The calculated IC₅₀ values were 30.56 μg/mL for Hep-G2 and 56.1 μg/mL for MCF-7, indicating stronger antiproliferative activity against liver cancer cells compared with breast cancer cells.

The enhanced anticancer activity of ZnO–MnO BNC can be primarily attributed to oxidative stress-mediated mechanisms commonly associated with metal oxide nanomaterials. ZnO NPs are known to generate intracellular ROS, which induce oxidative damage to cellular macromolecules and disrupt mitochondrial membrane potential, ultimately triggering apoptosis pathways. Excess ROS production leads to lipid peroxidation, DNA fragmentation, and activation of caspase-dependent cell death signaling (Rasmussen et al., 2010; Manke et al., 2013). Differences in sensitivity between Hep-G2 and MCF-7 cells may be related to variations in cellular metabolism, membrane composition, and antioxidant capacity. Hep-G2 cells appear more susceptible to ZnO–MnO BNC exposure, as reflected by the lower IC₅₀ value, suggesting enhanced NPs uptake or increased oxidative stress response in hepatic cancer cells. Similar variations in NPs’ sensitivity among cancer cell types have been widely reported and are influenced by cellular redox status and mitochondrial activity (Rasmussen et al., 2010). Importantly, when compared with the cytotoxicity results obtained for the WI-38 normal cell line (IC50 = 168.34 μg/mL), ZnO–MnO BNC demonstrated markedly higher toxicity toward cancer cells than normal cells. This selective anticancer behavior indicates a favorable therapeutic index and highlights the potential of the synthesized NC as a promising candidate for anticancer applications with reduced damage to healthy tissues.

#### Isolation and identification of *A. baumannii*

**Figure 7:**
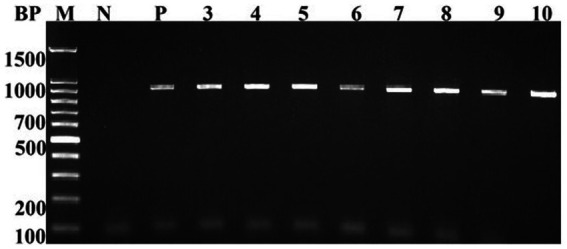
Representative agarose gel of the PCR amplicons showing 988-bp band specific for the *bla_oxa-51_*-lik_e_ intrinsic gene of *A. baumannii*. Wells: M, DNA marker;(N), negative control;(P), positive control using *A. baumannii* ATCC 17978; and 1–8,(A)*. baumannii* clinical isolates (Ab_1_–Ab_8_).

Eleven *A. baumannii* isolates, designated Ab_1_-Ab_11_, grow on MacConkey agar as non-lactose fermenting, round, mucoid, and opaque colonies. Also, the growth findings showed their ability to grow at 42 °C and 44 °C, and the TSI exhibited yellow butt and slant. Microscopically, the isolates revealed Gram-negative reaction with small non-motile capsulated bacilli in a single or paired arrangement. Biochemically, the obtained isolates exhibited positive citratase, ornithine decarboxylase, and catalase results and negative oxidase, tryptophanase, methyl red, and Voges-Proskauer results. Confirmed *A. baumannii* diagnosis was genotypically established based on the observation of the PCR amplicon of 988-bp specific *bla*_oxa-51-like_ intrinsic gene (Figure 7).

#### *Acinetobacter baumannii* resistance patterns

The antimicrobial susceptibility pattern of *A. baumannii* isolates is presented in Table 2. All isolates showed consistent resistance to ampicillin, amoxicillin, and amoxicillin/clavulanate. Resistance to cefotaxime varies, with some isolates sensitive and others resistant, while chloramphenicol generally retains activity except in one isolate from a burn wound. Sensitivity to tetracycline was more variable; most isolates are sensitive, but resistance appears in burn and wound samples. Resistance to ciprofloxacin and levofloxacin was observed in several isolates, particularly from wound and burn specimens. Colistin, amikacin, and gentamicin remain largely effective, although some resistance is noted, especially in wound isolates. Trimethoprim resistance is frequent, rendering it less reliable for treatment. Notably, the isolates from sputum tend to be more sensitive to certain antimicrobial agents compared to those from wounds or burns, which display wide resistance, underscoring the therapeutic challenges posed by *A. baumannii* in nosocomial infection.

**Table 2:** Antimicrobial resistance profile of *A. baumannii* isolates from clinical specimens.

*A. baumannii* Code	Specimen type	Antimicrobial agents											
		Ampicillin	Amoxicillin	Cefotaxime	Amoxicillin /Clavulanate	Chloramphenicol	Tetracycline	Ciprofloxacin	Levofloxacin	Colistin	Amikacin	Gentamicin	Trimethoprim
Ab_1_	Sputum	R*	R	S*	R	S	S	R	S	S	S	S	R
Ab_2_	Wound	R	R	R	R	S	S	R	S	S	R	R	R
Ab_3_	Sputum	R	R	S	R	S	R	S	S	S	S	S	R
Ab_4_	Sputum	R	R	S	R	S	S	R	S	S	S	S	R
Ab_5_	Burn	R	R	R	R	S	R	R	R	S	S	S	R
Ab_6_	Sputum	R	R	S	R	S	S	R	S	S	S	S	S
Ab_7_	Wound	R	R	R	R	S	S	R	R	S	R	R	R
Ab_8_	Wound	R	R	R	R	S	R	R	R	S	R	R	R
Ab_9_	Sputum	R	R	R	R	S	S	R	R	S	S	S	R
Ab_10_	Burn	R	R	R	R	R	R	R	R	S	S	R	R
Ab_11_	Sputum	R	R	R	R	R	S	S	S	S	S	R	R

*R: resistant and S: sensitive.

#### Qualitative screening of ZnO-MnO BNC activity

**Figure 8:**
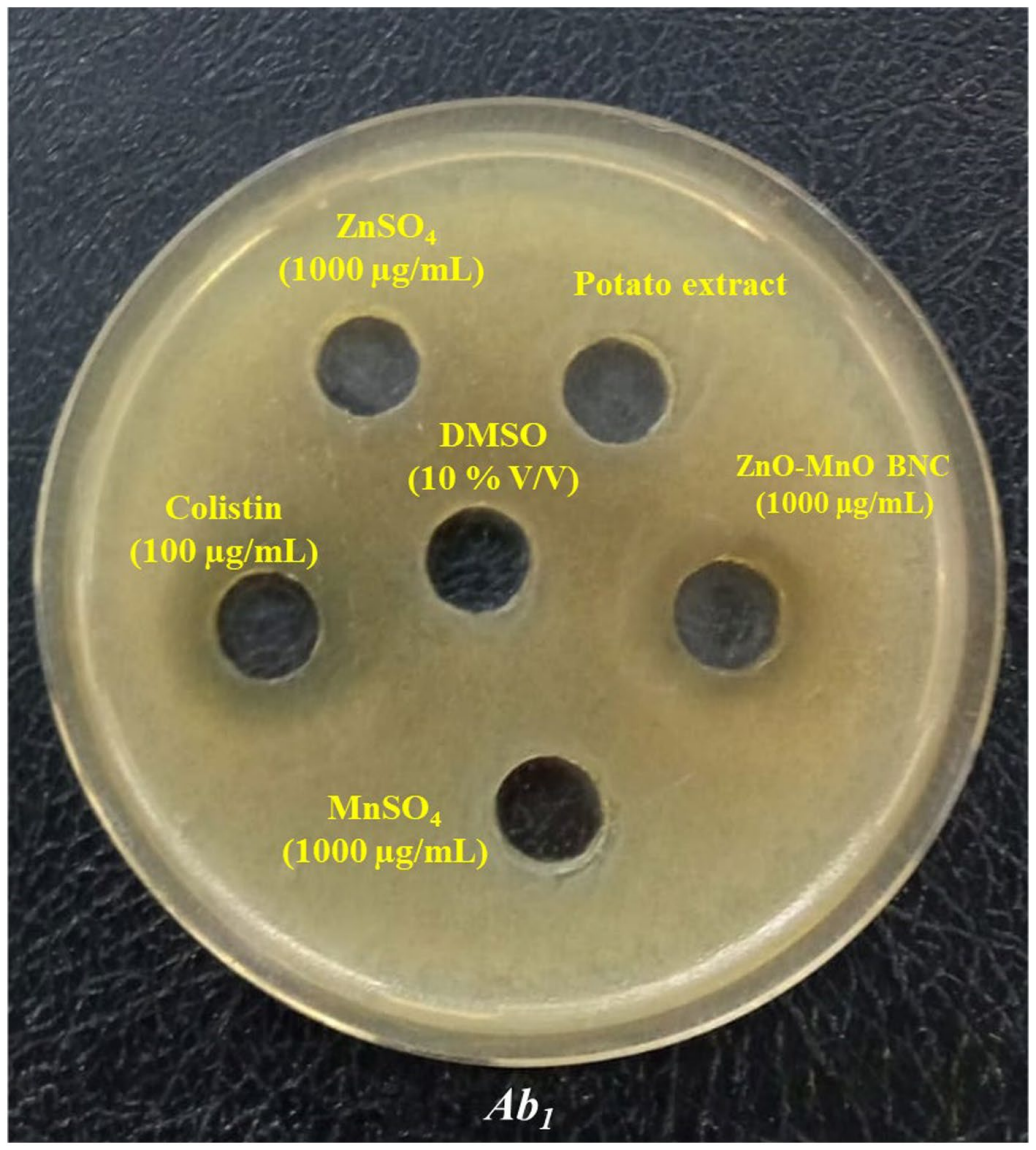
Antibacterial activity of ZnO–MnO BNC against *A. baumannii* (Ab_1_) isolates using a well diffusion assay.

The well diffusion assay results underscore the *in vitro* antibacterial efficacy of the biosynthesized ZnO–MnO BNC against a panel of 11 MDR *A. baumannii* clinical isolates and the reference strain ATCC 17978 (Figure 8). As detailed in Table 3, the tested ZnO–MnO BNC produced IZD ranging from 10.33 ± 0.58 mm to 20.33 ± 0.58 mm, reflecting variable responsiveness among the tested strains. In comparison, colistin, a reference standard antimicrobial agent, consistently yielded broader IZD ranging from 17.67 ± 0.58 mm to 22.67 ± 0.58 mm, affirming its established potency. The relative performance of the BNC, PI values were calculated against colistin for each isolate, revealing a spectrum of activity from 58.49 to 98.39%. This heterogeneity indicates a strain-specific interaction with the BNC, with isolates such as Ab_3_ and Ab_9_ exhibiting near-parallel inhibitory profiles to colistin, whereas others, notably Ab_8_, demonstrated reduced susceptibility.

**Table 3:** Qualitative antibacterial activity of ZnO–MnO BNC against *A. baumannii* isolates using a well diffusion assay.

Specimens	Isolates	Inhibition zone diameter (mm)		PI*
		ZnO-MnO BNC 1,000 μg/mL	Colistin* 50 μg/mL	
Sputum	Ab_1_	17 ± 1	19.67 ± 0.58	86.44
Wound	Ab_2_	12.33 ± 0.58	18.67 ± 0.58	66.07
Sputum	Ab_3_	18 ± 1	18.33 ± 0.58	98.18
Sputum	Ab_4_	14.67 ± 0.58	20.33 ± 0.58	72.13
Burn	Ab_5_	13.33 ± 0.58	21.33 ± 0.58	62.5
Sputum	Ab_6_	14.67 ± 0.58	19.67 ± 0.58	74.58
Wound	Ab_7_	13.33 ± 0.58	18.33 ± 0.58	72.73
Wound	Ab_8_	10.33 ± 0.58	17.67 ± 0.58	58.49
Sputum	Ab_9_	20.33 ± 0.58	20.67 ± 0.58	98.39
Burn	Ab_10_	12.33 ± 0.58	18.33 ± 0.58	67.27
Sputum	Ab_11_	16.33 ± 0.58	19.33 ± 0.58	84.48
–	Ab_17978_	15.67 ± 0.58	22.67 ± 0.58	69.12

*Colistin: used as positive control and PI: percent inhibition.

Gudkov et al. (2021) reviewed the broad-spectrum antibacterial effect of ZnO NPs, emphasizing their ability to generate ROS, disrupt bacterial membranes, and induce oxidative stress (Gudkov et al., 2021). Similarly, Fadwa et al. (2021) demonstrated that ZnO NPs enhanced the activity of antimicrobial agents such as ciprofloxacin and colistin against MDR *A. baumannii*, with inhibition zones reaching up to 22 mm (Shokrollahi et al., 2021). A more recent study by Rezaei et al. (2021) showed that levofloxacin-loaded ZnO NPs achieved significant inhibition against MDR strains, comparable to standard antimicrobial agents (Ahmed et al., 2025). Compared to these reports, the current data suggest that ZnO–MnO BNC offer comparable or superior efficacy, particularly in isolates Ab_3_ and Ab_9_, where inhibition zones nearly matched those of colistin. The incorporation of manganese may enhance NPs stability and ROS generation, contributing to the observed antimicrobial potency. Also, our findings are consistent with a study reporting remarkable antimicrobial efficacy for the biogenically capped ZnO NPs. Curcumin-capped ZnO NPs exhibited superior antibacterial activity against *K. pneumoniae* SGGDG (1.095 cm inhibition zone) and antifungal activity against *Exophiala* sp. SGVMC (0.764 cm inhibition zone), outperforming bare ZnO NPs and Teak-coated counterparts (Gupta et al., 2025).

One-way ANOVA revealed statistically significant differences in IZD among the isolates treated with ZnO–MnO BNC (*p* < 0.05). The mean inhibition zone for ZnO–MnO BNC was 15.14 ± 2.78 mm, compared to 19.63 ± 1.43 mm for colistin. Despite colistin showing slightly higher overall efficacy, ZnO–MnO BNC achieved PI values above 70% in 8 out of 12 strains, indicating consistent antimicrobial performance.

#### Inhibitory concentrations of ZnO–MnO BNC

**Figure 9:**
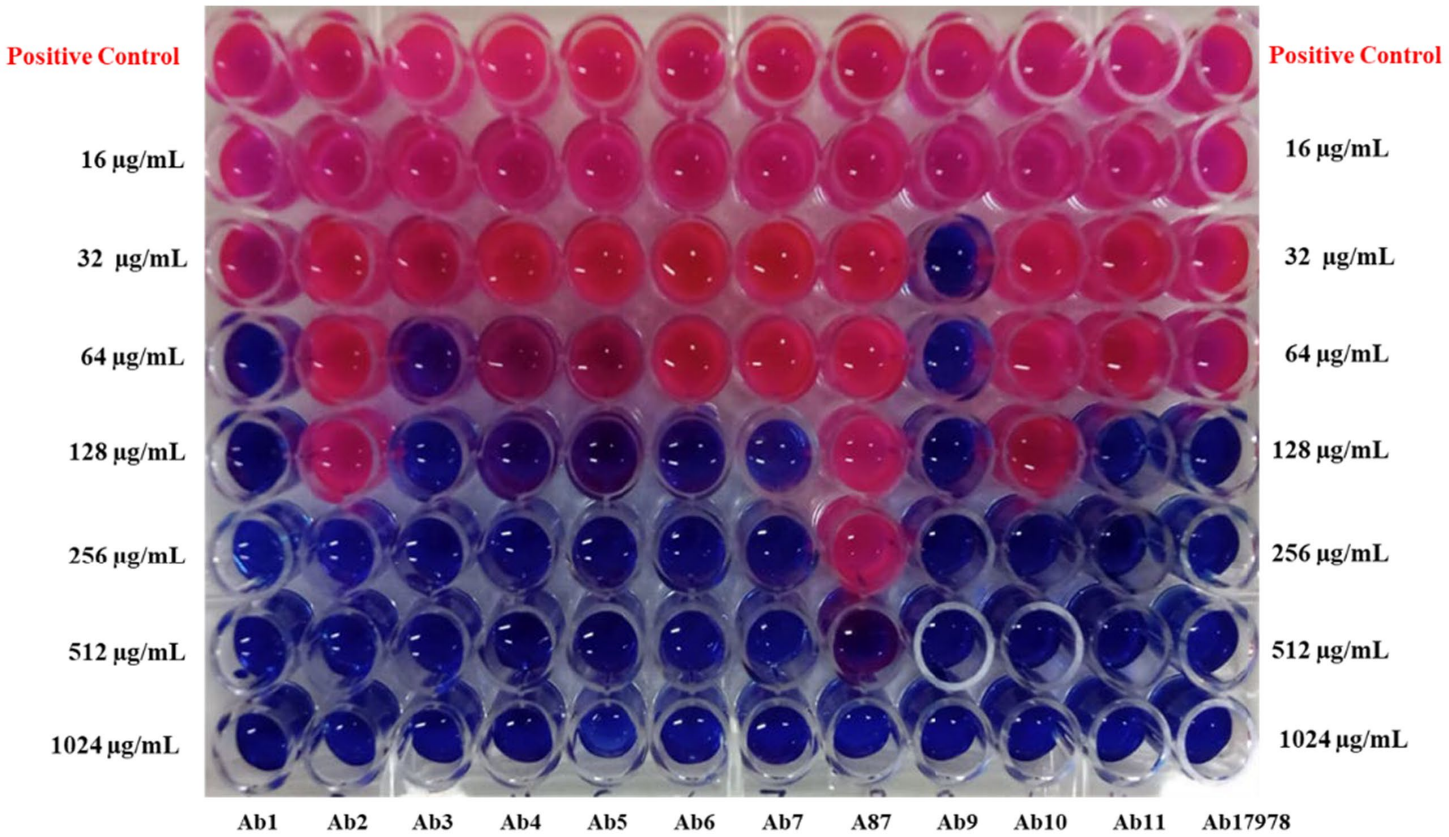
Broth microdilution assay shows the MIC of ZnO–MnO BNC against *A. baumannii* clinical isolates (Ab_1_–Ab_11_) and *A. baumannii* ATCC 17978 (Ab_17978_).

**Figure 10:**
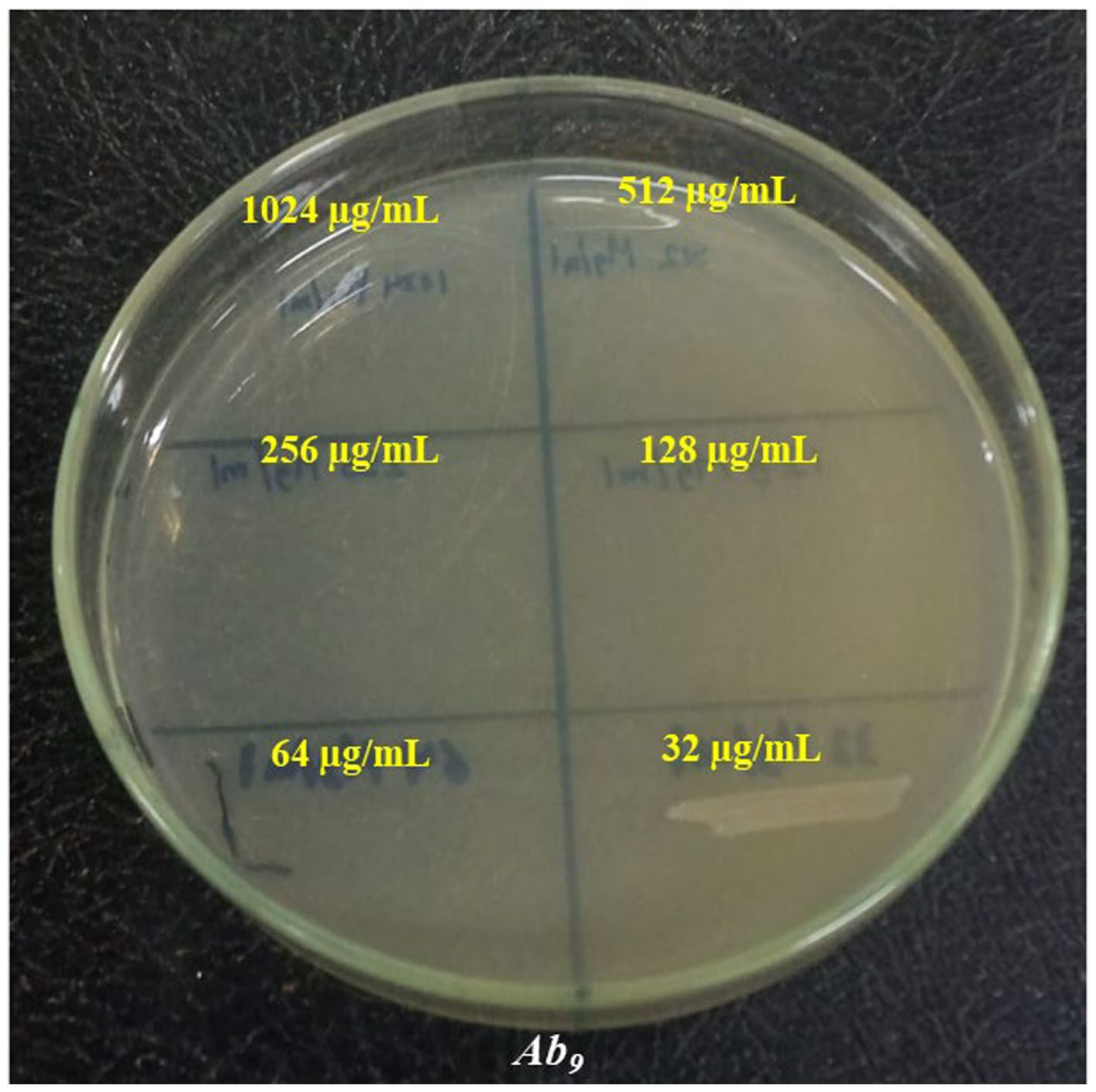
Minimum bactericidal concentration of ZnO–MnO BNC against *A. baumannii* (Ab_9_) clinical isolate.

Both the microdilution assays in Figure 9 illustrate the inhibitory concentrations of ZnO–MnO BNC against 11 *A. baumannii* clinical isolates and one reference strain (Table 4). The MIC values spanned from 32 μg/mL for Ab_9_ to 512 μg/mL, in the case of Ab_8_, while the MBC test (Figure 10) determined its values in the range of 64 μg/mL to 1,024 μg/mL. The MIC_50_ values, indicative of median inhibitory potency, varied from 24.541 to 188.13 μg/mL. The calculated tolerance index ranged between 1 and 4, with seven isolates falling in the strong bactericidal range (tolerance index ≤ 2). Notably, isolates Ab_3_ and Ab_9_ exhibited the lowest MIC and MIC_50_ values, suggesting heightened sensitivity to ZnO–MnO BNC. Burn and wound isolates generally required higher concentrations for inhibition and killing, consistent with their MDR profiles.

**Table 4:** Inhibitory concentrations of ZnO–MnO BNC against *A. baumannii*.

Specimens	*A. baumannii* isolates	Inhibitory concentrations (μg/mL)			TI*	Antibacterial effect
		MIC	MBC	MIC_50_		
Sputum	Ab_1_	64	256	50.004	4	Bactericidal
Wound	Ab_2_	256	512	157.222	2	Strong bactericidal
Sputum	Ab_3_	64	64	46.962	1	
Sputum	Ab_4_	128	256	77.612	2	
Burn	Ab_5_	128	512	64.371	4	Bactericidal
Sputum	Ab_6_	128	512	67.462	4	
Wound	Ab_7_	128	512	69.466	4	
Wound	Ab_8_	512	1,024	123.79	2	Strong bactericidal
Sputum	Ab_9_	32	64	19.541	2	
Burn	Ab_10_	256	1,024	188.13	4	Bactericidal
Sputum	Ab_11_	128	256	83.173	2	Strong bactericidal
-	Ab_17978_	128	256	82.493	2	

*TI: tolerance index (MBC/MIC ratio).

Recent studies corroborate the antimicrobial efficacy of ZnO-based nanomaterials against *A. baumannii*. Nan et al. (2024) reviewed the broad-spectrum antibacterial properties of nanoscale ZnO, highlighting its membrane-disruptive and oxidative stress-inducing mechanisms (Nan et al., 2024). A 2025 study by Ahmed et al. demonstrated that levofloxacin-loaded ZnO NPs synthesized via *Streptococcus mitis* biomass exhibited enhanced activity against MDR *A. baumannii*, with MIC values as low as 32 μg/mL (Ahmed et al., 2025). Similarly, biosynthesized ZnO NPs tested by El-Sayed et al. (2024) showed MICs ranging from 64 to 256 μg/mL against carbapenem-resistant *A. baumannii*, aligning with the current dataset (Selim et al., 2024). In another similar study, ZnO/chitosan/amoxicillin NC demonstrated superior antimicrobial activity against MDR *P. aeruginosa* clinical isolates and biofilms, achieving MIC and MBC values as low as 10 μg/mL and ≤ 100% biofilm inhibition at 150 μg/mL, with a favorable safety profile (IC50 = 292 μg/mL on Vero cells) (El-Zahed et al., 2026).

These findings reinforce the observed bactericidal potency of ZnO–MnO BNC, particularly against sputum-derived isolates, and validate their potential as alternative therapeutics in resistant infections.

A one-way ANOVA comparing MIC values across specimen types (sputum, wound, and burn) revealed a statistically significant difference (*p* < 0.05), with sputum isolates showing lower mean MICs (mean = 92 μg/mL) than wound (mean = 256 μg/mL) and burn isolates (mean = 192 μg/mL). Tukey’s *post hoc* test confirmed significant pairwise differences between sputum and wound (*p* = 0.03) and sputum and burn (*p* = 0.04), but not between wound and burn (*p* = 0.67). Similarly, MIC_50_ values were significantly lower in sputum isolates (mean = 61.29 μg/mL) compared to wound (mean = 116.16 μg/mL) and burn (mean = 126.25 μg/mL). These results suggest that sputum-derived *A. baumannii* isolates are more susceptible to ZnO–MnO BNC, potentially due to lower biofilm density or reduced efflux activity.

#### Biofilm inhibitory assay of ZnO–MnO BNC

**Figure 11:**
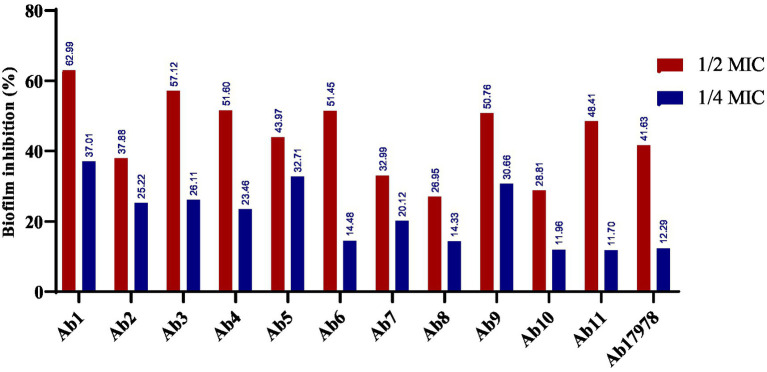
Biofilm inhibitory effect of ZnO–MnO BNC at subinhibitory concentrations (½MIC and ¼MIC) against *A. baumannii* clinical isolates (Ab_1_–Ab_11_) and *A. baumannii* ATCC 17978 (Ab_17978_).

The biosynthesized ZnO–MnO BNC revealed notable antibiofilm activity against *A. baumannii* isolates from diverse clinical sources (Figure 11). The biosynthesized BNC demonstrated dose-dependent biofilm inhibition against *A. baumannii* isolates, with ½MIC concentrations yielding significantly higher suppression than ¼MIC across all specimen types. At ½MIC, inhibition ranged from 26.95 to 62.99%, with the highest effect observed in Ab_1_ isolated from sputum. In contrast, ¼MIC concentrations showed reduced efficacy, ranging from 11.7 to 37.01%. Sputum-derived isolates generally exhibited stronger inhibition, suggesting possible specimen-specific susceptibility. The Ab_17978_ standard strain showed moderate inhibition (41.63% at ½MIC), validating the NPs’ broad-spectrum potential.

Recent studies corroborate the efficacy of ZnO-based NPs against biofilm-forming *A. baumannii*. Naskar et al. reported that ZnO NPs achieved up to 60% biofilm inhibition at sub-MIC levels, aligning closely with the current findings (Naskar et al., 2023). Similarly, chitosan–ZnO NC demonstrated dose-dependent antibiofilm activity, with enhanced effects at higher concentrations (Jabbar et al., 2025). A novel formulation using levofloxacin-loaded ZnO NPs also showed significant biofilm suppression, attributed to synergistic antimicrobial and gene expression modulation (Ahmed et al., 2025). Compared to these, the ZnO-MnO BNC in this study offers comparable or superior inhibition, particularly at ½MIC, suggesting that bimetallic synergy may enhance antibiofilm potency. Likewise, these observations align with recent findings, which reported that biogenically capped ZnO NPs, specifically those functionalized with curcumin, exhibit potent antimicrobial properties and effectively disrupt biofilm-forming microbial communities (Lahiri et al., 2022).

A paired t-test was conducted to compare biofilm inhibition at ½MIC versus ¼MIC across all isolates. The mean inhibition at ½MIC was 45.13% (±10.6), significantly higher than 21.38% (±8.3) at ¼MIC (*p* < 0.001), indicating a statistically significant dose-dependent effect.

#### ZnO–MnO BNC kill kinetics

**Figure 12:**
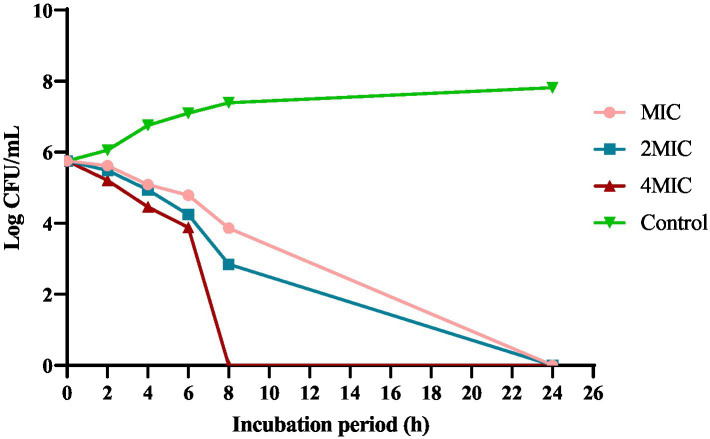
Time-kill kinetics of ZnO–MnO BNC against Ab_9_ isolate over 24 h.

The biosynthesized ZnO–MnO BNC exhibited a concentration-dependent bactericidal effect against Ab_9_ isolate, with complete growth inhibition achieved at 4MIC within 8 h of incubation (Table 5). The time-kill kinetics revealed a marked reduction in viable cell count over 24 h (Figure 12). The control untreated group showed exponential growth, reaching approximately 9 log CFU/mL. In contrast, the MIC, 2MIC, and 4MIC treatments progressively suppressed bacterial proliferation. Notably, the 4MIC condition achieved complete bacterial eradication by the eighth hour, indicating rapid and potent bactericidal activity. The 2MIC and MIC concentrations also reduced bacterial load significantly, though less rapidly, with residual growth observed beyond 12 h. These findings underscore the efficacy of ZnO–MnO BNC in disrupting *A. baumannii* viability in a dose- and time-dependent manner.

**Table 5:** Time kill assay of ZnO–MnO BNC against Ab_9_ clinical isolate.

Incubation period (h.)	Log CFU of Ab_9_ isolate			
	MIC (μg/mL)	2MIC (μg/mL)	4MIC (μg/mL)	Control
0 H	5.756	5.756	5.756	5.756
2 H	5.623	5.491	5.21	6.061
4 H	5.086	4.944	4.459	6.756
6 H	4.792	4.246	3.881	7.1
8 H	3.863	2.845	0	7.396
24 H	0	0	0	7.82

Recent investigations support the antimicrobial potential of ZnO–based nanomaterials against MDR *A. baumannii*. Kakian et al. demonstrated that ZnO NPs reduced planktonic cell viability, with time-kill assays showing significant log reductions at 2MIC and 4MIC levels (Kakian et al., 2024). Similarly, a study using levofloxacin-loaded ZnO NPs reported complete bacterial killing within 6–8 h at 4MIC, consistent with the current findings (Ahmed et al., 2025). These results suggest that bimetallic formulations, such as ZnO–MnO BNC, may enhance antimicrobial efficacy through synergistic ion release and oxidative stress mechanisms. Compared to monometallic ZnO NPs, the dual-metal approach appears to accelerate bacterial clearance, particularly in highly sensitive strains like Ab_9_.

Statistical analysis shows that the interaction between concentration and incubation time was statistically significant (*F* = 42.7, *p* < 0.001), indicating that bacterial reduction was both time- and dose-dependent. *Post hoc* Tukey’s test confirmed that 4MIC significantly differed from MIC and 2MIC at each time point beyond 4 h (*p* < 0.01). Additionally, linear regression analysis showed a strong negative correlation between BNC concentration and log CFU/mL (R^2^ = 0.93), reinforcing the dose–response relationship. These results validate the bactericidal potency of ZnO–MnO BNC and support their potential as a therapeutic alternative against resistant *A. baumannii* strains.

#### Combined ZnO-MnO BNC interaction with cefotaxime

**Figure 13:**
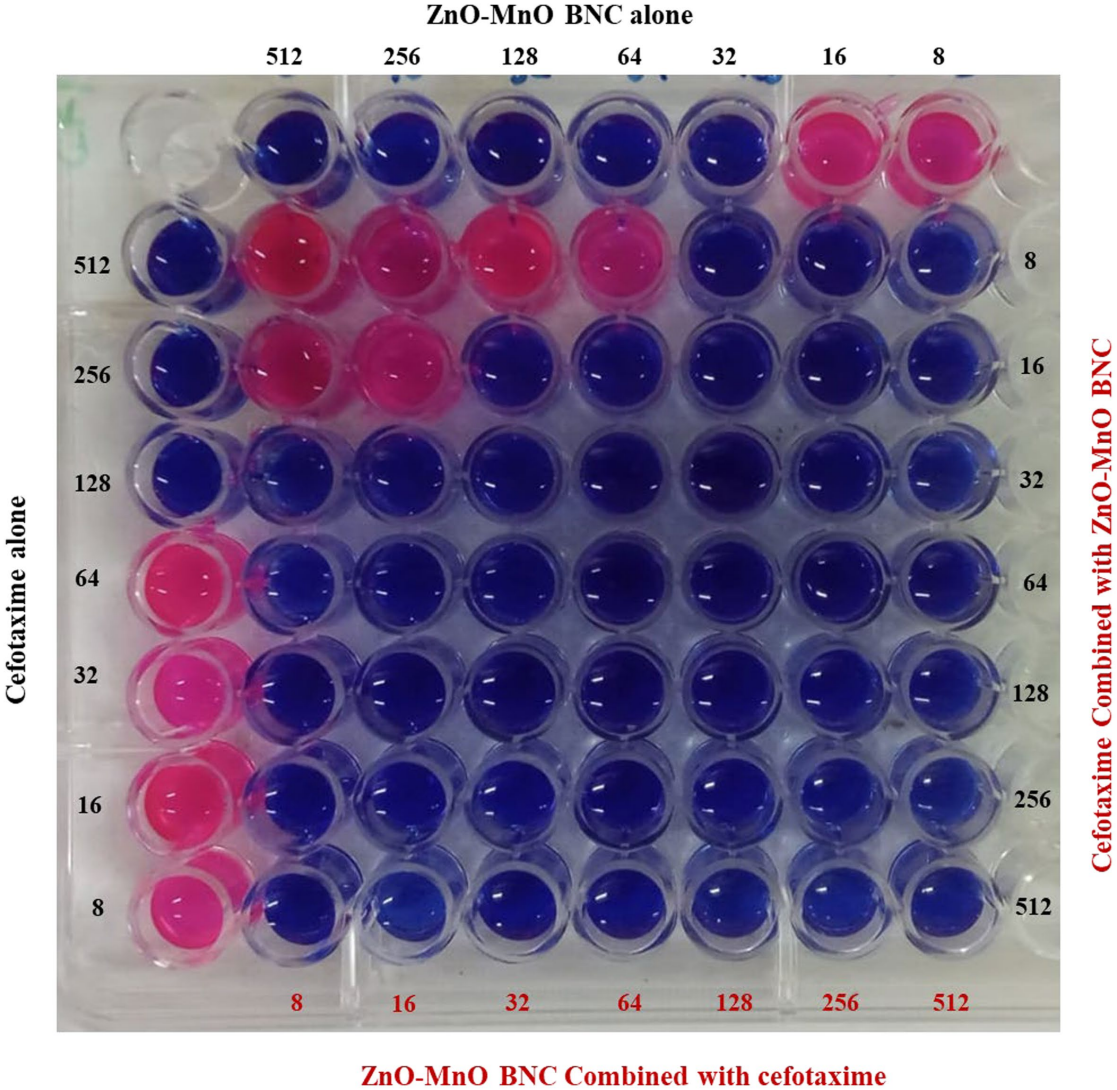
Checkerboard assay results showing the synergistic interaction between ZnO–MnO BNC and cefotaxime against *Ab1_7978_* using resazurin method-based broth microdilution assay. Yellow square represents the combination panel.

The checkerboard assay results evaluate the combined antimicrobial effect of ZnO–MnO BNC and cefotaxime against *A. baumannii* ATCC 17978 (Figure 13). The MICs of cefotaxime and ZnO–MnO BNC alone were 32 μg/mL and 128 μg/mL, respectively. When combined, the MICs were reduced to 8 μg/mL for cefotaxime and 32 μg/mL for ZnO–MnO BNC. The FIC values for both agents were 0.25, yielding a combined FICi of 0.5. According to standard interpretive criteria, an FICi ≤ 0.5 indicates synergism. These results confirm that the combination of ZnO–MnO BNC and cefotaxime exhibits a synergistic effect, enhancing antibacterial efficacy against the tested strain.

**Figure 14:**
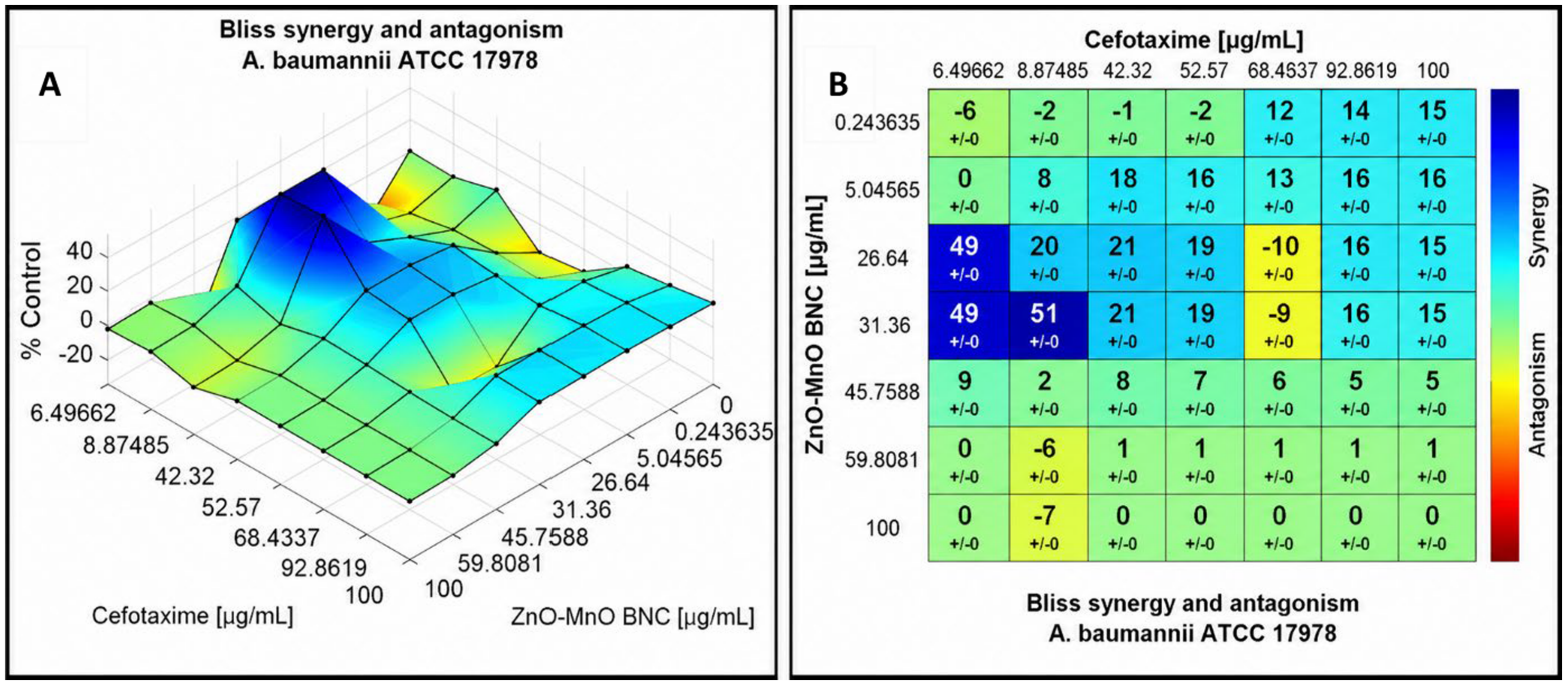
Synergistic interaction between ZnO–MnO BNC and cefotaxime against *A. baumannii* ATCC 17978, analyzed using Combenefit software. **(A)** Surface Bliss and **(B)** Matrix Bliss collectively demonstrates enhanced antibacterial efficacy when both agents are combined. Positive synergy scores and elevated inhibition zones indicate that ZnO–MnO BNC potentiate the activity of cefotaxime, surpassing the effects of either agent alone.

The combined antimicrobial activity of ZnO–MnO BNC and cefotaxime was also evaluated against *A. baumannii* ATCC 17978 using Combenefit software (Figure 14). The Loewe synergy heatmap revealed positive interaction scores at multiple concentration combinations, particularly in the mid-range of both agents, indicating enhanced bacterial inhibition beyond additive effects. The Bliss independence contour plot further confirmed synergy, with elevated response zones where both agents were co-administered. The Highest Single Agent (HSA) surface plot showed that the combination outperformed either agent alone, with a marked reduction in bacterial viability. The 3D interaction surfaces across all models consistently demonstrated that ZnO–MnO BNC potentiated the activity of cefotaxime, suggesting a cooperative mechanism of action.

The observed synergy between ZnO–MnO BNC and cefotaxime reflects a promising combinatorial strategy against *A. baumannii*, a pathogen known for its MDR and biofilm-forming capacity. The enhanced efficacy may be attributed to the membrane-disruptive properties of the BNC, which facilitate increased antibiotic penetration and intracellular access. These findings align with recent reports by Al-Gariaa et al., who documented synergistic effects between ZnO–Ag NC and *β*-lactam antimicrobial agent against resistant *A. baumannii* strains, with synergy scores exceeding +10 in Bliss and Loewe models (Al-Gariaa et al., 2023). Similarly, Shokrollahi et al. demonstrated that ZnO NPs enhanced the bactericidal action of cefotaxime and ciprofloxacin against clinical isolates, with significant reductions in MIC values and biofilm density (Shokrollahi et al., 2021). Likewise, Gomaa et al. highlighted the synergistic antimicrobial efficacy of the Ag NPs/ZnO QDs/nitazoxanide composite against MDR pathogens (Gomaa et al., 2024).

The current data reinforce the utility of metal oxide NPs as adjuvants in antimicrobial therapy, particularly when targeting Gram-negative pathogens with compromised outer membranes. The synergy observed in this study suggests that ZnO–MnO BNC may disrupt membrane integrity or interfere with efflux mechanisms, thereby amplifying the intracellular concentration of cefotaxime. This dual-action approach could mitigate resistance development and improve therapeutic outcomes in clinical settings.

Quantitative synergy was assessed using Combenefit software across three models: Loewe, Bliss, and HSA. Mean synergy scores were extracted from the interaction matrices and compared using one-way ANOVA. The analysis revealed a statistically significant difference in synergy scores across models (*p* < 0.0001).

#### Protein leakage effect of ZnO-MnO BNC

The protein leakage assay revealed a progressive increase in extracellular protein concentration in response to escalating doses of ZnO–MnO BNC. At ¼MIC (8 μg/mL), the leaked protein reached 9.02 ± 0.059 μg/mL, corresponding to 13.05% leakage. This value rose to 17.5 ± 0.094 μg/mL (25.32%) at ½MIC and peaked at 50.77 ± 0.49 μg/mL (73.46%) at 2MIC (64 μg/mL). The MIC concentration (32 μg/mL) induced 39.98 ± 0.094 μg/mL leakage, equivalent to 57.85%. The PBS-treated control showed negligible leakage (0.37 ± 0.034 μg/mL, 0.54%), while the positive control treated with Triton X–100 yielded maximal leakage (69.11 ± 0.78 μg/mL, 100%). These findings demonstrate a concentration-dependent disruption of the tested bacterial membrane integrity by ZnO–MnO BNC, as shown in Table 6.

**Table 6:** Protein leakage from Ab_9_ clinical isolate treated with ZnO–MnO BNC.

Tested materials	Concentration	Leaked protein	
		(μg/mL)	(%)
ZnO-MnO BNC	¼MIC (8 μg/mL)	9.02 ± 0.059	13.05
	½MIC (16 μg/mL)	17.5 ± 0.094	25.32
	MIC (32 μg/mL)	39.98 ± 0.094	57.85
	2MIC (64 μg/mL)	50.77 ± 0.49	73.46
Triton X–100 Positive control	1%	69.11 ± 0.78	100
PBS Negative control		0.37 ± 0.034	0.54

The observed increase in protein leakage with rising NC concentration suggests that ZnO–MnO BNC compromises the cytoplasmic membrane of *A. baumannii*, leading to efflux of intracellular proteins. This membrane-disruptive effect is consistent with the known oxidative and mechanical stress induced by metal oxide NPs. Comparable findings were reported by Hassan and Sahib, who demonstrated that biosynthesized ZnO NPs caused significant protein leakage in *A. baumannii* isolates, with leakage levels exceeding 70% at 2MIC (Hassan and Sahib, 2025). Similarly, a study by Abdelaliem et al. showed that ZnO–Ag hybrid NPs induced dose-dependent membrane damage in MDR *A. baumannii*, confirmed by elevated protein and nucleic acid leakage (Abdelaliem et al., 2023). Protein leakage may be due to cell membrane disruption, and ZnO NPs can directly interact with the bacterial cell membrane, leading to its damage and subsequent leakage of intracellular contents (Yang et al., 2024). This physical disruption compromises the integrity of the cell, allowing essential components like proteins, DNA, and other cellular material to escape. Electrolyte leakage is a direct indicator of cell membrane damage and has been observed with ZnO NPs (Haidri et al., 2024).

The statistical analysis revealed a statistically significant difference in mean protein leakage values (*p* < 0.0001). *Post hoc* Tukey’s test confirmed that all treated groups (¼MIC to 2MIC) exhibited significantly higher leakage than the PBS control (*p* < 0.001), and that leakage at 2MIC was significantly greater than at MIC and ½MIC (*p* < 0.01). These results validate the dose-dependent effect of ZnO–MnO BNC on membrane permeability.

#### Morphological deterioration effect of ZnO–MnO BNC

**Figure 15:**
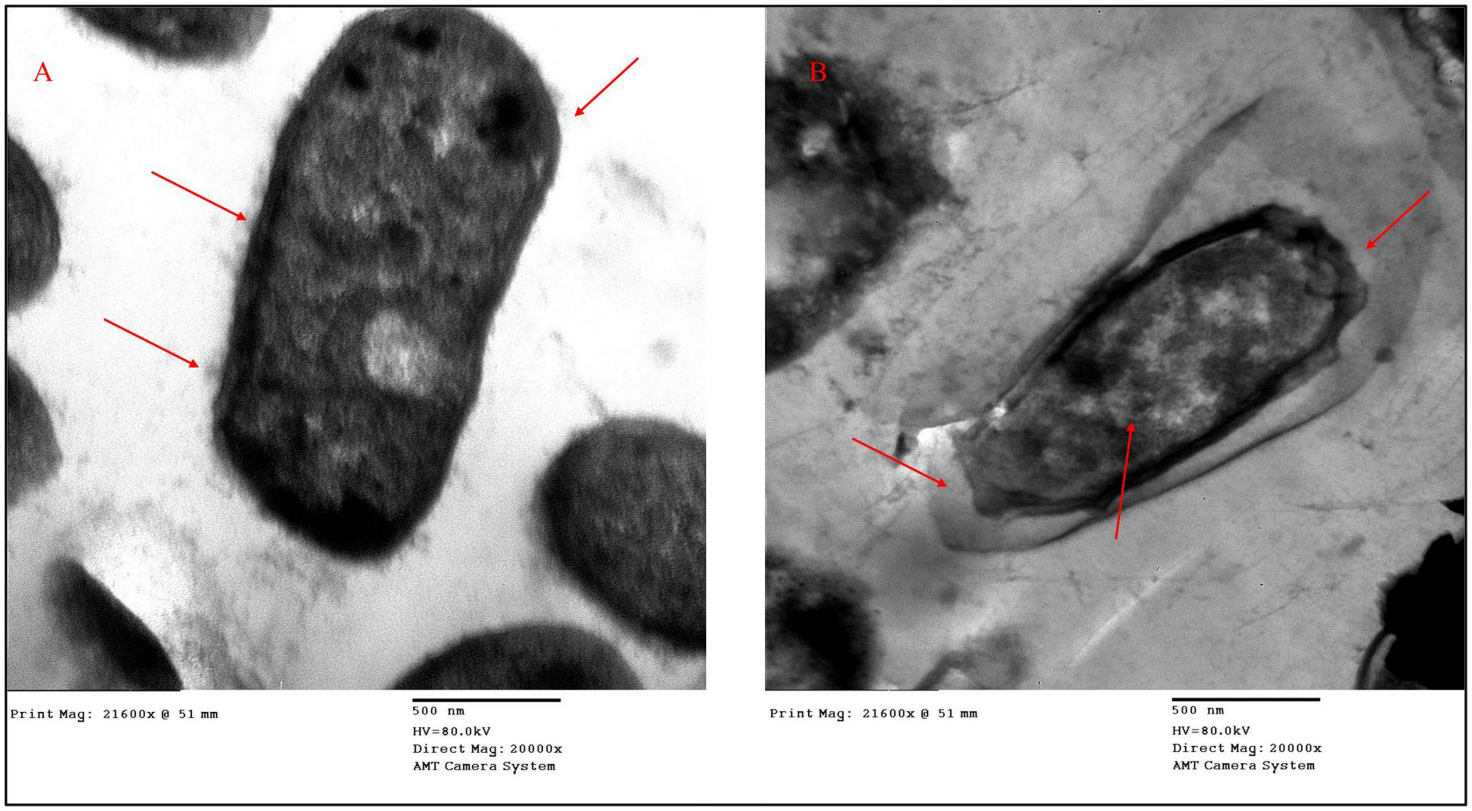
Transmission electron micrographs of *A. baumannii* strain 9 show: **(A)** Intact cell morphology with a smooth outer membrane and dense cytoplasmic content; **(B)** Cells treated with ZnO–MnO BNC (16 μg/mL) showing disrupted cell wall integrity, irregular membrane contours, and accumulation of extracellular material. Red arrows show significant ultrastructural changes, such as membrane deterioration and possible zones of NC interaction.

Transmission electron microscope images of *A. baumannii*-9 are shown at a magnification of 21,600×. The rod-shaped bacteria in untreated cells have a smooth, intact outer membrane and a dense interior architecture, both of which are traits of a healthy physiological state. However, bacterial cells treated with ZnO–MnO BNC at a concentration of 16 μg/mL show significant structural changes, irregular membrane contours, and damaged cell wall structures, as indicated by the red arrows; these could be indicators of exopolysaccharide release or biofilm breakdown. The presence of a translucent halo around the treated cell, which suggests membrane leakage or NC contact, is consistent with antimicrobial stress responses (Figure 15).

The morphological alteration seen in ZnO–MnO NP-treated cells is consistent with earlier research on bacterial disruption caused by NPs. Our results are compatible with Selim et al. (2024), who reported that biosynthesized ZnO NPs produced cell wall rupture, cytoplasmic condensation, and membrane leakage in carbapenem-resistant *A. baumannii* (Selim et al., 2024). In addition, Tiwari et al. (2018) further elucidated that ZnO NPs exert antibacterial effects via ROS generation, lipid peroxidation, and protein denaturation, leading to visible structural collapse under TEM (Tiwari et al., 2018). Our study extends this understanding by incorporating manganese, which may enhance oxidative stress and metal ion synergy.

It is crucial to recognize that the synergistic interaction may involve additional and complementary pathways, even though the membrane-disruptive characteristics of ZnO–MnO BNC, as demonstrated by the protein leakage assay and TEM imaging, probably represent a primary mechanism underlying the observed synergy with cefotaxime. One of the most important and well-studied mechanisms causing MDR in *A. baumannii* is efflux pump-mediated resistance; the AdeABC, AdeFGH, and AdeIJK efflux systems are especially important in lowering intracellular accumulation of various antimicrobial agents with consequent resistance (Coyne et al., 2011). Metal oxide NPs have been proposed to potentially interfere with efflux pump activity through direct interaction with membrane-embedded transporter proteins, disruption of the proton motive force required for efflux pump function, and generation of oxidative stress that damages the energy-dependent efflux machinery (Hemeg, 2017). Future research employing specialized assays, such as efflux pump inhibitor combination assays and gene expression studies of efflux pump-encoding genes, should examine the role of efflux pump inhibition and other resistance routes.

## Conclusion

This study successfully demonstrated the green synthesis of a ZnO–MnO BNC using potato peel extract as a cost-effective and environmentally friendly technique. Comprehensive physicochemical examination verified a stable, crystalline biphasic NC consisting of hexagonal ZnO and cubic MnO phases with an average particle size of 35 nm and hydrodynamic diameter of 38 nm. The biosynthesized ZnO–MnO BNC showed selective biological performance with high anticancer activity against Hep-G2 and MCF-7 cancer cell lines (IC_50_ = 30.56 and 56.1 μg/mL, respectively) and comparatively mild toxicity against normal WI-38 cells (IC_50_ = 168.34 μg/mL). Additionally, the BNC showed potent antibacterial action against clinical isolates of MDR *A. baumannii*, with MIC values ranging from 32 to 512 μg/mL and inhibition zones between 10.33 and 20.33 mm. Significant antibiofilm activity was demonstrated, with inhibition reaching 62.99% at ½MIC. Time-kill kinetics demonstrated complete bacterial eradication at 4MIC in 8 h, while the protein leakage experiment confirmed concentration-dependent membrane breakdown, with leakage reaching 73.46% at 2MIC. Its synergistic interaction with cefotaxime (FICI = 0.5) further demonstrates its potential as an adjuvant therapy agent that can enhance the effectiveness of conventional antibiotics. These findings suggest that ZnO-MnO BNC is a promising alternative for biomedical and antimicrobial applications, necessitating further *in vivo* investigation and clinical translation investigations.

## Data Availability

The original contributions presented in the study are included in the article/supplementary material, further inquiries can be directed to the corresponding authors.

## References

[ref1] Abdelaliem Y. F. Abdel-Baset T. A. N. Sayed A. R. M. Owis A. A. Ramadan M. F. Mohdaly A. (2023). Characterization of ZnO and Mn-doped ZnO nanoparticles and their antimicrobial activity. Rend. Fis. Acc. Lincei 34, 189–198. doi: 10.1007/s12210-022-01126-0, 30311153

[ref2] Abraham A. (2024). Understanding the Effect of Phytochemical Coated Silver Nanoparticles on Mammalian Cells and the Protein Interactions with the Surface Corona of These Nanoparticles. Melbourne: RMIT University.

[ref3] Adeyemi F. M. Akinlade E. A. Yusuf-Omoloye N. A. Ajigbewu O. H. Dare A. P. Wahab A. A. . (2025). Carbapenem-resistance in *Acinetobacter baumannii*: prevalence, antibiotic resistance profile and carbapenemase genes in clinical and hospital environmental strains. BMC Infect Dis 25:786. doi: 10.1186/s12879-025-11169-x, 40457246 PMC12128347

[ref4] Ahmed M. E. Al-Awadi A. Q. Mohamed H. S. (2025). Molecular biology insights into levofloxacin-loaded ZnO nanoparticles: a potent strategy against MDR *Acinetobacter baumannii*. RSC Adv. 15, 30189–30201. doi: 10.1039/D5RA03081A, 40874159 PMC12378608

[ref5] Al Abboud M. A. Mashraqi A. Qanash H. Gattan H. S. Felemban H. R. Alkorbi F. . (2024). Green biosynthesis of bimetallic ZnO@AuNPs with its formulation into cellulose derivative: biological and environmental applications. Bioresour. Bioprocess. 11:60. doi: 10.1186/s40643-024-00759-3, 38884830 PMC11183018

[ref6] Alamgeer Tahir M. Sarker M. R. Ali S. Ibraheem Hussian S. Ali S. . (2023). Polyaniline/ZnO hybrid nanocomposite: morphology, spectroscopy and optimization of ZnO concentration for photovoltaic applications. Polymers (Basel) 15:363. doi: 10.3390/polym15020363, 36679244 PMC9865263

[ref7] Al-Gariaa A. M. Elasala G. Ismail E. H. Khalil M. M. El-Sewify I. M. (2023). Photodegradation of antibacterial cefotaxime using Mn doped ZnO nanosphere. Inorg. Chem. Commun. 158:111434. doi: 10.1016/j.inoche.2023.111434, 38826717

[ref8] Balapure A. Dutta J. R. Ganesan R. (2024). Recent advances in semiconductor heterojunctions: a detailed review of the fundamentals of photocatalysis, charge transfer mechanism and materials. Royal Soc. Chem. 1, 43–69. doi: 10.1039/D3LF00126A

[ref9] Basak M. Rahman M. L. Ahmed M. F. Biswas B. Sharmin N. (2022). The use of X-ray diffraction peak profile analysis to determine the structural parameters of cobalt ferrite nanoparticles using Debye-Scherrer, Williamson-hall, Halder-Wagner and size-strain plot: different precipitating agent approach. J. Alloys Compd. 895:162694. doi: 10.1016/j.jallcom.2021.162694

[ref10] Bauer A. W. Kirby W. M. Sherris J. C. Turck M. (1966). Antibiotic susceptibility testing by a standardized single disk method. Am J Clin Pathol 45, 493–496. doi: 10.1093/ajcp/45.4_ts.493, 5325707

[ref11] Bellio P. Fagnani L. Nazzicone L. Celenza G. (2021). New and simplified method for drug combination studies by checkerboard assay. MethodsX 8:101543. doi: 10.1016/j.mex.2021.101543, 34754811 PMC8563647

[ref9001] CLSI (2023). Performance Standards for Antimicrobial Susceptibility Testing. CLSI supplement M100. 33rd ed. Wayne, PA: Clinical and Laboratory Standards Institute.

[ref12] Coyne S. Courvalin P. Périchon B. (2011). Efflux-mediated antibiotic resistance in *Acinetobacter* spp. Antimicrob. Agents Chemother. 55, 947–953. doi: 10.1128/AAC.01388-10, 21173183 PMC3067115

[ref13] Dolai J. Mandal K. Jana N. R. (2021). Nanoparticle size effects in biomedical applications. ACS Appl. Nano Mater. 4, 6471–6496. doi: 10.1021/acsanm.1c00987

[ref14] Eissa M. E. (2024). Resistance mechanisms and treatment burdens for *A. baumannii* infections: current challenges and future opportunities. J. Biochem. Microbiol. 1, 1–1.

[ref15] Elashmawi I. S. Al-Muntaser A. A. (2021). Influence of Co3O4 nanoparticles on the optical, and electrical properties of CMC/PAM polymer: combined FTIR/DFT study. J. Inorg. Organomet. Polym. Mater. 31, 2682–2690. doi: 10.1007/s10904-021-01956-9

[ref16] El-Batal A. I. Al-Shammari B. M. El-Sayyad G. S. Rizk S. H. Abdelaziz A. M. Nofel M. M. . (2024). Gum Arabic-assisted biomass synthesis of bimetallic ZnO-CuO nanoparticles using gamma rays for controlling potato post-harvest tuber rots-causing Alternaria solani: towards improving food safety. Biomass Convers. Biorefinery 14, 31877–31894. doi: 10.1007/s13399-023-04836-z

[ref17] Elkady F. M. Badr B. M. Hashem A. H. Abdulrahman M. S. Abdelaziz A. M. Al-Askar A. A. . (2024). Unveiling the *Launaea nudicaulis* (L.) hook medicinal bioactivities: phytochemical analysis, antibacterial, antibiofilm, and anticancer activities. Front Microbiol 15:1454623. doi: 10.3389/fmicb.2024.1454623 39421554 PMC11484093

[ref18] Elkady F. M. Badr B. M. Saied E. Hashem A. H. Abdulrahman M. S. Alkherkhisy M. M. . (2025). Mycosynthesis of zinc oxide nanoparticles using Mucor racemosus with their antimicrobial, antibiofilm, anticancer and antioxidant activities. Sci Rep. 15:18772. doi: 10.1038/s41598-025-03421-w, 40436936 PMC12120078

[ref19] El-Moslamy S. H. Abd-Elhamid A. I. Fawal G. E. (2024). Large-scale production of myco-fabricated ZnO/MnO nanocomposite using endophytic Colonstachys rosea with its antimicrobial efficacy against human pathogens. Sci. Rep. 14:935. doi: 10.1038/s41598-024-51398-9, 38195769 PMC10776836

[ref20] El-Sayed A. F. Aboulthana W. M. Sherief M. A. El-Bassyouni G. T. Mousa S. M. (2024). Synthesis, structural, molecular docking, and in vitro biological activities of cu-doped ZnO nanomaterials. Sci. Rep 14:9027. doi: 10.1038/s41598-024-59088-2, 38641640 PMC11031592

[ref21] El-Zahed M. M. Radwan Y. M. Abou-Dobara M. I. (2026). Optimized green zinc oxide/chitosan/amoxicillin nanocomposite against *Pseudomonas aeruginosa*. Appl. Microbiol. Biotechnol. 110:119. doi: 10.1007/s00253-026-13768-3, 41876748 PMC13018007

[ref22] Espina A. Sanchez-Cortes S. Jurašeková Z. (2022). Vibrational study (Raman, SERS, and IR) of plant gallnut polyphenols related to the fabrication of Iron gall inks. Molecules 27:279. doi: 10.3390/molecules27010279 35011511 PMC8746386

[ref9002] Fadwa A. O. Albarag A. M. Alkoblan D. K. Mateen A. (2021). Determination of synergistic effects of antibiotics and Zno NPs against isolated E. Coli and A. Baumannii bacterial strains from clinical samples. Saudi J. Biol. Sci. 28, 5332–5337. 34466112 10.1016/j.sjbs.2021.05.057 PMC8380994

[ref23] Girigoswami A. Deepika B. Pandurangan A. K. Girigoswami K. (2024). Preparation of titanium dioxide nanoparticles from *Solanum Tuberosum* peel extract and its applications. Artif. Cells Nanomed. Biotechnol. 52, 59–68. doi: 10.1080/21691401.2023.2301068, 38214666

[ref24] Girma A. Alamnie G. Bekele T. Mebratie G. Mekuye B. Abera B. . (2024). Green-synthesised silver nanoparticles: antibacterial activity and alternative mechanisms of action to combat multidrug-resistant bacterial pathogens: a systematic literature review. Green Chem. Lett. Rev. 17:2412601. doi: 10.1080/17518253.2024.2412601

[ref25] Gomaa I. Aleid G. El-Moslamy S. H. Alshammari A. Al-Marshedy S. Alshammary F. . (2024). Synergistic efficacy of ZnO quantum dots, ag NPs, and nitazoxanide composite against multidrug-resistant human pathogens as new trend of revolutionizing antimicrobial treatment. Discover Nano 19:164. doi: 10.1186/s11671-024-04085-7, 39361062 PMC11450118

[ref26] Gudkov S. V. Burmistrov D. E. Serov D. A. Rebezov M. B. Semenova A. A. Lisitsyn A. B. J. F. I. P. (2021). A mini review of antibacterial properties of ZnO nanoparticles. Front. Phys. 9:641481. doi: 10.3389/fphy.2021.641481 PMC8300809 34356805

[ref27] Gupta S. Kashyap M. Kumar V. Jain P. Vinayak V. Joshi K. B. (2018). Peptide mediated facile fabrication of silver nanoparticles over living diatom surface and its application. J. Mol. Liq. 249, 600–608. doi: 10.1016/j.molliq.2017.11.086

[ref28] Gupta S. Kumar V. Yadav N. Dagar R. Kalyankar R. Gupta K. (2025). Enhanced antimicrobial activity of biogenic zinc oxide (ZnO) nanoparticles. ChemistrySelect 10:e01732. doi: 10.1002/slct.202501732

[ref29] Haidri I. Ishfaq A. Shahid M. Hussain S. Shahzad T. Shafqat U. . (2024). Enhancement of antioxidants’ enzymatic activity in the wheat crop by *Shewanela* sp. mediated zinc oxide nanoparticles against heavy metals contaminated wastewater. J. Soil Sci. Plant Nutr. 24, 7068–7089. doi: 10.1007/s42729-024-02025-z

[ref30] Hakim A. A. N. Rashid A. R. A. Arsad N. Surani A. H. (2020). Zinc oxide thin film synthesized by sol-gel method. Solid State Phenom. 307, 51–57. doi: 10.4028/www.scientific.net/ssp.307.51

[ref31] Hasanin M. S. Hashem A. H. Al-Askar A. A. Haponiuk J. Saied E. (2023). A novel nanocomposite based on mycosynthesized bimetallic zinc-copperoxide nanoparticles, nanocellulose and chitosan: characterization, antimicrobial and photocatalytic activities. Electron. J. Biotechnol. 65, 45–55. doi: 10.1016/j.ejbt.2023.05.001

[ref32] Hashem A. H. El-Sayyad G. S. (2024). Antimicrobial and anticancer activities of biosynthesized bimetallic silver-zinc oxide nanoparticles (ag-ZnO NPs) using pomegranate peel extract. Biomass Convers. Biorefinery 14, 20345–20357. doi: 10.1007/s13399-023-04126-8

[ref33] Hassan N. Sahib R. (2025). Biosynthesis of zinc oxide nanoparticles produced from *Penicillium chrysogenum* against *Acinetobacter baumannii* isolated from renal failure patients. Egypt. J. Med. Microbiol. 34:1657. doi: 10.21608/ejmm.2025.386061.1657

[ref34] Hemeg H. A. (2017). Nanomaterials for alternative antibacterial therapy. Int. J. Nanomedicine 12, 8211–8225. doi: 10.2147/IJN.S132163, 29184409 PMC5689025

[ref35] Hernández-Rodríguez Y. M. Garcia-Tejeda Y. V. Baños-López E. Cigarroa-Mayorga O. E. (2025). Semi-automatic system for ZnO nanoflakes synthesis via electrodeposition using bioinspired neuro-fuzzy control. Biomimetics (Basel) 10:712. doi: 10.3390/biomimetics10100712, 41149242 PMC12562137

[ref36] Hoseinpour V. Ghaemi N. (2018). Novel ZnO–MnO2–Cu2O triple nanocomposite: facial synthesis, characterization, antibacterial activity and visible light photocatalytic performance for dyes degradation-a comparative study. Mater. Res. Express 5:085012. doi: 10.1088/2053-1591/aad2c6

[ref37] Hussein N. M. Mortazavi-Drazkola S. (2025). Biosynthesized ZnO-based bimetallic nanocomposite for anticancer, antimicrobial, and photocatalytic applications. Bioprocess Biosyst. Eng. 48, 913–926. doi: 10.1007/s00449-025-03150-4, 40121599

[ref38] Ijaz N. Bashir S. Ikram A. Zafar A. Ul Ain H. B. Ambreen S. . (2024). Valorization of potato peel: a sustainable eco-friendly approach. CyTA J. Food 22:2306951. doi: 10.1080/19476337.2024.2306951

[ref39] Ioset J.-R. Brun R. Wenzler T. Kaiser M. Yardley V. (2009). Drug Screening for Kinetoplastids Diseases. A Training Manual for Screening in Neglected Diseases. DNDi and Pan-Asian Screening Network, Swiss Tropical And Public Health Institute.

[ref40] Jabbar Y. S. A. Gazi U. Ahmed M. E. Engineering P. (2025). Investigation of the effects of chitosan-zinc nanoparticles Ch-ZnO NPs on phenotypic biofilm formation of *Acinetobacter baumannii* and cytotoxicity assay. Iraqi J. Chem. Petroleum Eng 26, 77–88. doi: 10.31699/IJCPE.2025.1.8

[ref41] Jang Y. Lee S.-H. Kim N.-K. Ahn C. H. Rittmann B. E. Park H.-D. J. W. R. (2023). Biofilm characteristics for providing resilient denitrification in a hydrogen-based membrane biofilm reactor. Water Res. 231:119654. doi: 10.1016/j.watres.2023.119654, 36702020

[ref42] Kainat S. Gilani S. R. Asad F. Khalid M. Z. Khalid W. Ranjha M. M. . (2022). Determination and comparison of phytochemicals, phenolics, and flavonoids in *Solanum lycopersicum* using FTIR spectroscopy. Food Anal. Methods 15, 2931–2939. doi: 10.1007/s12161-022-02344-w

[ref43] Kakian F. Arasteh N. Mirzaei E. Motamedifar M. (2024). Study of MIC of silver and zinc oxide nanoparticles, strong and cost-effective antibacterial against biofilm-producing *Acinetobacter baumannii* in Shiraz, Southwest of Iran. BMC Infect Dis 24:593. doi: 10.1186/s12879-024-09471-1, 38886629 PMC11181610

[ref44] Kalakonda P. Mandal P. Laxmi Mynepally S. Bashipangu A. Kethavath A. Khanam S. J. . (2024). Comparison of multi-metallic nanoparticles-alternative antibacterial agent: understanding the role of their antibacterial properties. J. Inorg. Organomet. Polym. Mater. 34, 2203–2218. doi: 10.1007/s10904-023-02960-x

[ref45] Kaur J. N. Singh N. Smith N. M. Klem J. F. Cha R. Lang Y. . (2024). Next generation antibiotic combinations to combat pan-drug resistant *Klebsiella pneumoniae*. Sci. Rep. 14:3148. doi: 10.1038/s41598-024-53130-z, 38326428 PMC10850076

[ref46] Khanal S. Karimi K. Majumdar S. Kumar V. Verma R. Bhatia S. K. . (2024). Sustainable utilization and valorization of potato waste: state of the art, challenges, and perspectives. Biomass Convers. Biorefinery 14, 23335–23360. doi: 10.1007/s13399-023-04521-1

[ref47] Khorsand Zak A. Hashim A. M. (2024). Optical properties analysis of gelatin-based prepared co, Ni, and Mn-doped ZnO nanoparticles in infrared and UV–Vis region using Kramers-Kronig methods. Ceram. Int. 50, 51501–51508. doi: 10.1016/j.ceramint.2024.10.068

[ref48] Kinoan C. M. Katas H. J. R. A. (2025). Sustainable production and antibacterial efficacy of silver nanoparticles on cellulose nanofibers from mushroom waste. RSC Adv. 15, 19726–19740. doi: 10.1039/d5ra02087e, 40503294 PMC12152727

[ref49] Kirubakaran D. Wahid J. B. A. Karmegam N. Jeevika R. Sellapillai L. Rajkumar M. . (2026). A comprehensive review on the green synthesis of nanoparticles: advancements in biomedical and environmental applications. Biomed. Mater. Devices 4, 388–413. doi: 10.1007/s44174-025-00295-4

[ref50] Lahiri D. Ray R. R. Sarkar T. Upadhye V. J. Ghosh S. Pandit S. . (2022). Anti-biofilm efficacy of green-synthesized ZnO nanoparticles on oral biofilm: *in vitro* and *in silico* study. Front. Microbiol. 13:9390. doi: 10.3389/fmicb.2022.939390, 36262331 PMC9574224

[ref51] Mandal A. K. Katuwal S. Tettey F. Gupta A. Bhattarai S. Jaisi S. . (2022). Current research on zinc oxide nanoparticles: synthesis, characterization, and biomedical applications. Nano 12:3066. doi: 10.3390/nano12173066, 36080103 PMC9459703

[ref52] Manke A. Wang L. Rojanasakul Y. (2013). Mechanisms of nanoparticle-induced oxidative stress and toxicity. Biomed. Res. Int. 2013:942916. doi: 10.1155/2013/942916, 24027766 PMC3762079

[ref53] Martínez-Vargas B. L. Cruz-Ramírez M. Díaz-Real J. A. Rodríguez-López J. L. Bacame-Valenzuela F. J. Ortega-Borges R. . (2019). Synthesis and characterization of n-ZnO/p-MnO nanocomposites for the photocatalytic degradation of anthracene. J. Photochem. Photobiol. A Chem. 369, 85–96. doi: 10.1016/j.jphotochem.2018.10.010

[ref54] Mathur P. Kumawat M. Nagar R. Singh R. Daima H. K. (2024). Tailoring metal oxide nanozymes for biomedical applications: trends, limitations, and perceptions. Anal. Bioanal. Chem. 416, 5965–5984. doi: 10.1007/s00216-024-05416-4, 39009769

[ref55] Nan J. Chu Y. Guo R. Chen P. (2024). Research on the antibacterial properties of nanoscale zinc oxide particles comprehensive review. Front. Mater. 11:1449614. doi: 10.3389/fmats.2024.1449614

[ref56] Naskar A. Cho H. Kim K.-S. J. J. O. C. S. (2023). Black phosphorus-based ZnO-ag nanocomposite for antibacterial activity against tigecycline-resistant *Acinetobacter baumannii*. J. Compos. Sci. 7:423. doi: 10.3390/jcs7100423

[ref57] Pani S. Singh S. K. Mohapatra B. K. (2019). Synthesis and characterization of MnO Nano-particles using thermal plasma technique. Trans. Indian Inst. Metals 72, 65–71. doi: 10.1007/s12666-018-1461-2

[ref58] Pierce V. M. Bhowmick T. Simner P. (2023). Guiding antimicrobial stewardship through thoughtful antimicrobial susceptibility testing and reporting strategies: an updated approach in 2023. J Clin Microbiol 61:e0007422. doi: 10.1128/jcm.00074-22 37768094 PMC10662363

[ref59] Pradeep M. Kruszka D. Kachlicki P. Mondal D. Franklin G. (2022). Uncovering the phytochemical basis and the mechanism of plant extract-mediated eco-friendly synthesis of silver nanoparticles using ultra-performance liquid chromatography coupled with a photodiode Array and high-resolution mass spectrometry. ACS Sustain. Chem. Eng. 10, 562–571. doi: 10.1021/acssuschemeng.1c06960

[ref60] Rajmichael R. Hemavathy N. Mathimaran A. Pandian C. J. Kingsley J. D. Subramanian G. . (2025). Whole genome sequencing characterization and comparative genome analysis of *Acinetobacter baumannii* JJAB01: a comprehensive insights on antimicrobial resistance and virulence genotype. Microb Pathog. 199:107224. doi: 10.1016/j.micpath.2024.107224, 39675438

[ref61] Rasmussen J. W. Martinez E. Louka P. Wingett D. G. (2010). Zinc oxide nanoparticles for selective destruction of tumor cells and potential for drug delivery applications. Expert Opin. Drug Deliv. 7, 1063–1077. doi: 10.1517/17425247.2010.502560, 20716019 PMC2924765

[ref9003] Rezaei M. R. Sayadi M. H. Ravankhah N. (2021). Photocatalytic degradation of amoxicillin and levofloxacin from aqueous solutions using Ag/ZnO.

[ref62] Rizvi M. Gerengi H. Gupta P. (2022). “Functionalization of nanomaterials: synthesis and characterization,” in Functionalized Nanomaterials for Corrosion Mitigation: Synthesis, Characterization, and Applications, (New York: American Chemical Society), 1–26.

[ref63] Saied E. Basher N. S. Badr B. M. Elkady F. M. Mostafa A. G. Ibrahim N. A. . (2026). Eco-friendly biosynthesis of manganese oxide-silver bimetallic nanoparticles using *Cucumis melo* peel extract: characterization, antioxidant, antimicrobial, and antiviral activities. Bioresour. Bioprocess. 13:19. doi: 10.1186/s40643-025-00987-1, 41615612 PMC12858710

[ref64] Saravanakumar K. Sakthivel P. Sankaranarayanan R. K. (2022). Influence of Sn4+ ion on band gap tailoring, optical, structural and dielectric behaviors of ZnO nanoparticles. Spectrochim. Acta A Mol. Biomol. Spectrosc. 267:120487. doi: 10.1016/j.saa.2021.120487, 34689004

[ref65] Selim S. Abdelghany T. M. Almuhayawi M. S. Nagshabandi M. K. Tarabulsi M. K. Elamir M. Y. M. . (2025a). Biosynthesis and activity of Zn-MnO nanocomposite in vitro with molecular docking studies against multidrug resistance bacteria and inflammatory activators. Sci. Rep. 15:2032. doi: 10.1038/s41598-024-85005-8, 39814844 PMC11735634

[ref66] Selim S. Almuhayawi M. S. Saddiq A. A. Alruhaili M. H. Saied E. Sharaf M. H. . (2025b). Synthesis of novel MgO-ZnO nanocomposite using *Pluchea indica* leaf extract and study of their biological activities. Bioresour. Bioprocess. 12:33. doi: 10.1186/s40643-025-00848-x, 40220116 PMC11993530

[ref67] Selim M. I. Sonbol F. I. El-Banna T. E. Negm W. A. Elekhnawy E. (2024). Antibacterial and wound healing potential of biosynthesized zinc oxide nanoparticles against carbapenem-resistant *Acinetobacter baumannii*: An *in vitro* and *in vivo* study. Microbial Cell Factories 23:281. doi: 10.1186/s12934-024-02538-3, 39415253 PMC11484456

[ref68] Shalini V. Shanmugam R. Manigandan P. Sciences B. (2024). Cytoplasmic leakage and protein leakage analysis of *Ocimum gratissimum* stem extract-mediated silver nanoparticles against wound pathogens. J Pharm Bioallied Sci 16, S1354–S1359. doi: 10.4103/jpbs.jpbs_578_23, 38882859 PMC11174165

[ref69] Sheema Zafar S. Khan S. Jamal Q. Uzair M. Akbar S. . (2025). Green synthesis, biological potential, and semiconducting properties of MnO:ZnO bimetallic nanocomposites. J. Inorg. Organomet. Polym. Mater. 35, 6688–6708. doi: 10.1007/s10904-025-03689-5

[ref70] Shi J. Cheng J. Liu S. Zhu Y. Zhu M. (2024). *Acinetobacter baumannii*: an evolving and cunning opponent. Front Microbiol. 15:1332108. doi: 10.3389/fmicb.2024.1332108, 38318341 PMC10838990

[ref71] Shokrollahi B. Bafroee A. S. T. Saleh T. J. (2021). Effect of zinc oxide nanoparticles on loaded antibiotics against multidrug-resistant *Acinetobacter* spp. Avicenna J. Clin. Microbiol. Infect. 8, 51–56. doi: 10.34172/ajcmi.2021.10

[ref72] Singh H. Desimone M. F. Pandya S. Jasani S. George N. Adnan M. . (2023). Revisiting the green synthesis of nanoparticles: uncovering influences of plant extracts as reducing agents for enhanced synthesis efficiency and its biomedical applications. Int. J. Nanomedicine 18, 4727–4750. doi: 10.2147/IJN.S419369, 37621852 PMC10444627

[ref73] Somu P. Kannan U. Paul S, and Biotechnology (2019). Biomolecule functionalized magnetite nanoparticles efficiently adsorb and remove heavy metals from contaminated water. J. Chem. Technol. Biotechnol. 94, 2009–2022, doi: 10.1002/jctb.5984, .41531421

[ref74] Szczyglewska P. Feliczak-Guzik A. Nowak I. (2023). Nanotechnology–general aspects: a chemical reduction approach to the synthesis of nanoparticles. Molecules 28:4932. doi: 10.3390/molecules28134932 37446593 PMC10343226

[ref75] Tabassum N. Khan F. Jeong G.-J. Oh D. Kim Y. (2024). Antibiofilm and antivirulence activities of laminarin-gold nanoparticles in standard and host-mimicking media. Appl Microbiol Biotechnol 108:203. doi: 10.1007/s00253-024-13050-4, 38349556 PMC10864539

[ref76] Tasnim N. T. Ferdous N. Rumon M. M. H. Shakil M. S. (2023). The promise of metal-doped iron oxide nanoparticles as antimicrobial agent. ACS Omega 9, 16–32. doi: 10.1021/acsomega.3c06323, 38222657 PMC10785672

[ref77] Tiwari V. Mishra N. Gadani K. Solanki P. Shah N. Tiwari M. (2018). Mechanism of anti-bacterial activity of zinc oxide nanoparticle against carbapenem-resistant *Acinetobacter baumannii*. Front Microbiol 9:1218. doi: 10.3389/fmicb.2018.01218, 29928271 PMC5997932

[ref78] Tovar-Lopez F. J. (2023). Recent Progress in Micro- and nanotechnology-enabled sensors for biomedical and environmental challenges. Sensors 23:5406. doi: 10.3390/s23125406, 37420577 PMC10300794

[ref79] Tümer M. (2025). Ganoderma Lucidum Ekstraktından Mikosentez Yoluyla Metal Oksit Nanopartikül Sentezi ve Bazı Biyolojik Aktivitelerinin Belirlenmesi, bursa uludağ university.

[ref80] Tyutrina V. A. Sosedova L. M. Novikov M. A. (2024). Analysis of the genotoxicity of iron nanocomposite arabinogalactan using the DNA comet method. Hygiene Sanitation 103, 1251–1256. doi: 10.47470/0016-9900-2024-103-10-1251-1256

[ref81] Umar A. Ribeiro C. Al-Hajry A. Masuda Y. Hahn Y. B. (2009). Growth of highly c-Axis-oriented ZnO nanorods on ZnO/glass substrate: growth mechanism, structural, and optical properties. J. Phys. Chem. C 113, 14715–14720. doi: 10.1021/jp9045098

[ref82] Valcek A. Philippe C. Whiteway C. Robino E. Nesporova K. Bové M. . (2023). Phenotypic characterization and heterogeneity among modern clinical isolates of *Acinetobacter baumannii*. Microbiol. Spectrum 11:e03061-03022. doi: 10.1128/spectrum.03061-22, 36475894 PMC9927488

[ref83] Van De Loosdrecht A. Beelen R. Ossenkoppele G. Broekhoven M. Langenhuijsen M. (1994). A tetrazolium-based colorimetric MTT assay to quantitate human monocyte mediated cytotoxicity against leukemic cells from cell lines and patients with acute myeloid leukemia. J. Immunol. Methods 174, 311–320. doi: 10.1016/0022-1759(94)90034-5, 8083535

[ref84] Virzì N. F. Greco V. Stracquadanio S. Jasim A. Greish K. Diaz-Rodriguez P. . (2024). Berberine-styrene-co-maleic acid nanomicelles: unlocking opportunities for the treatment and prevention of bacterial infections. RSC Adv. 14:34066-34080. doi: 10.1039/d4ra04457f, 39469023 PMC11513620

[ref85] Wilson B. K. Prud'homme R. K. (2021). Nanoparticle size distribution quantification from transmission electron microscopy (TEM) of ruthenium tetroxide stained polymeric nanoparticles. J. Colloid Interface Sci. 604, 208–220. doi: 10.1016/j.jcis.2021.04.081, 34265681

[ref86] Xie Y. P. Yang Y. Wang G. Liu G. (2017). Oxygen vacancies promoted interfacial charge carrier transfer of CdS/ZnO heterostructure for photocatalytic hydrogen generation. J. Colloid Interface Sci. 503, 198–204. doi: 10.1016/j.jcis.2017.05.006, 28525827

[ref87] Yakoup A. Y. Kamel A. G. Elbermawy Y. Abdelsattar A. S. El-Shibiny A. J. S. R. (2024). Characterization, antibacterial, and cytotoxic activities of silver nanoparticles using the whole biofilm layer as a macromolecule in biosynthesis. Sci. Rep. 14:364. doi: 10.1038/s41598-023-50548-9, 38172225 PMC10764356

[ref88] Yang F. Li Y. Mu B. Wang Q. Song Y. Wang A. (2024). Insight into the synergistic antibacterial mechanism of bio ZnO nanoparticles synthesized from *Sophora japonica* flower buds extract. Inorganic Chem. Commun. 163:112341. doi: 10.1016/j.inoche.2024.112341

